# Coordination of Cyanobacterial Nitrate Assimilation and Photosynthesis by a Novel PsbO‐Interacting Protein PirN

**DOI:** 10.1002/advs.202518047

**Published:** 2026-03-20

**Authors:** Chengcheng Huang, Zhen Xiao, Haitao Ge, Gaojie Wang, Jinghui Dong, Yan Wang, Hang Yang, Xing Wang, Hui Gao, Zhongshu Wang, Huanling Yang, Yuanya Zhang, Xiahe Huang, Wu Xu, Weimin Ma, Wenqiang Yang, Yingchun Wang

**Affiliations:** ^1^ Institute of Genetics and Developmental Biology Chinese Academy of Sciences Beijing China; ^2^ Central Laboratory, Shanxi Province Cancer Hospital/Shanxi Hospital Affiliated to Cancer Hospital Chinese Academy of Medical Sciences/Cancer Hospital Affiliated to Shanxi Medical University Taiyuan Shanxi China; ^3^ University of Chinese Academy of Sciences Beijing China; ^4^ State Key Laboratory of Forage Breeding‐By‐Design and Utilization and Key Laboratory of Photobiology Institute of Botany Chinese Academy of Sciences Beijing China; ^5^ Department of Chemistry University of Louisiana at Lafayette Lafayette Louisiana USA; ^6^ College of Life Sciences Shanghai Normal University Shanghai China; ^7^ China National Botanical Garden Beijing China

**Keywords:** cyanobacteria, nitrate assimilation, photosynthesis, PirN, PsbO

## Abstract

Nitrogen assimilation relies on photosynthetically produced energy and reducing equivalents. Here, we identify a small protein, PsbO‐interacting regulator of nitrate assimilation (PirN), as a key coordinator of photosynthesis and nitrate assimilation in *Synechocystis* sp. PCC 6803 (*Synechocystis*). PirN specifically accumulates in nitrate‐grown cells and interacts with the photosystem II (PSII) subunit PsbO. Deletion of *pirN* (Δ*pirN*) under nitrate impairs growth, reduces PSII contents and oxygen evolution, and upregulates most nitrogen transport and assimilation proteins except the nitrate reductase NarB, mimicking nitrogen starvation response likely due to impaired nitrate reduction. PsbO expression is markedly reduced in both Δ*pirN* and Δ*narB* mutants. Complementation of *narB* or *psbO* in the Δ*pirN* mutant background largely rescued the growth defect of the mutant and led to increased expression of PsbO or NarB, respectively. These findings suggest that PirN is a nitrate‐inducible regulator that hierarchically modulates NarB and PsbO expression to couple nitrate assimilation with photosynthetic activity. This coordination likely safeguards redox homeostasis when nitrate assimilation or photosynthetic electron production is perturbed.

## Graphical abstract

PirN was identified as a previously uncharacterized PsbO‐interacting protein specifically induced in nitrate‐grown cyanobacteria. Loss of PirN leads to coordinated downregulation of PsbO and the nitrate reductase NarB, causing decreased PSII activity and impaired growth under nitrate conditions. Complementation with either PsbO or NarB largely restores growth, demonstrating that PirN is essential for coupling photosynthesis with nitrate assimilation.

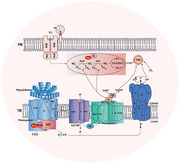

**Abbreviations**
2‐OG: 2‐oxoglutarate
BN‐PAGE: Blue native polyacrylamide gel electrophoresis
C/N: carbon/nitrogen
CBB: Coomassie Brilliant Blue
DCMU: 3‐(3,4‐dichlorophenyl)‐1,1‐dimethylurea
DDA: data‐dependent acquisition
DEPs: differentially expressed proteins
DIA: data‐independent acquisition
DTT: dithiothreitol
FA: formic acid
Fd: ferredoxin
GOGAT: glutamate‐oxoglutarate amidotransferase
GS: glutamine synthetase
IP‐MS: immunoprecipitation coupled with mass spectrometry
LC‐MS/MS: liquid chromatography–tandem mass spectrometry
NAGK: N‐acetylglutamate kinase
NarB: nitrate reductase
NirA: nitrite reductase
OD_730_
: optical density at 730 nm
OER: oxygen evolution rate
ORF: open reading frame
PC: plastocyanin
PirN: PsbO‐interacting regulator of nitrate assimilation
PM: plasma membrane
PMSF: phenylmethanesulfonyl fluoride
PSII: photosystem II
qPCR: quantitative real‐time PCR
SDS‐PAGE: sodium dodecyl sulfate‐polyacrylamide gel electrophoresis
*Synechocystis*
: *Synechocystis* sp. PCC 6803
TCA cycle: tricarboxylic acid cycle
TM: thylakoid membrane
WT: wild type

## 1 Introduction

Nitrogen and carbon are essential macronutrients that form the structural and functional foundation of life. Cyanobacteria are a group of photosynthetic bacteria that play a central role in global biogeochemical cycles of the two macronutrients. The optimal growth of cyanobacteria necessitates intricate coordination of nitrogen and carbon metabolism to maintain cellular homeostasis under fluctuating environmental conditions [1]. This coordination is essential not only for the synthesis of cellular building blocks such as amino acids, nucleotides, and other nitrogen‐containing molecules but also for the production of carbohydrates and other carbon‐rich metabolites [2].

Non‐diazotrophic cyanobacteria can take up combined nitrogen sources such as nitrate, nitrite, ammonium, and urea from their natural habitats [3, 4, 5]. Assimilation of these combined nitrogen requires carbon skeletons produced from CO_2_ fixation through the Calvin‐Benson cycle and the downstream tricarboxylic acid (TCA) cycle. Nitrogen assimilation and carbon metabolism are hence functionally interconnected and inherently coordinated. Disruption of one process can profoundly affect the other, as evidenced by nitrogen limitation, which induces differential expression not only of proteins involved in nitrogen assimilation [6, 7, 8, 9], but also those in carbon metabolism [10]. The interplay between nitrogen and carbon metabolism is governed by a complex regulatory network involving transcriptional regulators, post‐transcriptional modifications, and signaling metabolites such as 2‐oxoglutarate (2‐OG), ATP, and cyclic AMP [1, 11, 12]. Among combined nitrogen sources, nitrate is the most abundant in many natural habitats of cyanobacteria [13, 14]. Nitrate assimilation begins with its transport into the cell, typically via an ABC‐type transporter encoded by the *nrtABCD* operon [15]. Once inside, nitrate is reduced to nitrite and then to ammonium through reactions catalyzed by ferredoxin (Fd)‐dependent nitrate reductase (NarB) and nitrite reductase (NirA), respectively [15]. In these steps, reduced Fd serves as the electron donor, providing two electrons for the reduction of nitrate to nitrite and six electrons for the subsequent reduction of nitrite to ammonium. The resulting ammonium is subsequently incorporated into the carbon skeleton of 2‐OG, an intermediate of the TCA cycle, via the GS‐GOGAT cycle, which involves the enzymatic activities of glutamine synthetase (GS) and glutamate‐oxoglutarate amidotransferase (GOGAT) and consumes two additional electrons [16].

Nitrogen assimilation requires significant amounts of energy and reducing equivalents, particularly when nitrate is the nitrogen source. Assimilating a single nitrate molecule consumes 10 electrons as described above [17], mainly supplied by reducing agents such as photosynthetically reduced Fd. Under balanced growth conditions, most cyanobacteria maintain an optimal carbon/nitrogen (C/N) ratio of approximately 5:1 [1], reflecting the atomic ratio of carbon to nitrogen in glutamate, the product of the GS‐GOGAT cycle. Considering that the fixation of one molecule of CO_2_ requires four electrons, the ratio of electrons required for nitrate assimilation to CO_2_ fixation is approximately 1:2, assuming a C/N ratio of 5:1. This suggests that the electron demands for these two processes are comparable, and perturbation of either process could disrupt cellular homeostasis.

In cyanobacteria, the electrons required for nitrogen assimilation and CO_2_ fixation are ultimately derived from photosynthesis. The photolysis of two H_2_O molecules during the light reaction generates four electrons and releases one molecule of O_2_ [18]. The efficient generation and allocation of these electrons to CO_2_ fixation and nitrogen assimilation require tight regulatory coordination, and evidence suggests the existence of mechanisms that synchronize these physiological processes. For instance, nitrate assimilation has been shown to enhance oxygen evolution and photosynthetic electron transport compared with ammonium [17], as the assimilation of a single nitrate molecule demands eight more electrons that are ultimately generated from water photolysis. For a similar reason, nitrate and nitrite reductases have been proposed to act as an electron sink, preventing the over‐reduction of photosynthetically reduced electron acceptors [19]. Additionally, studies on PsbO, a subunit of the oxygen‐evolving complex in PSII, indicate its involvement in regulating the C/N balance in the cyanobacterium *Synechocystis* [20, 21]. Although key proteins involved in coordinating carbon metabolism and nitrogen assimilation, such as PII and PirC [22, 23], have been identified, the presence of a similar regulator coordinating nitrogen assimilation and photosynthetic electron production remains unknown.

Recently, we identified proteins near the luminal side of PSII in *Synechocystis* using an APEX2‐based proximity labeling approach with PsbO as the bait [24]. *Synechocystis* was the first cyanobacterium to have its genome fully sequenced and is highly transformable, making it an ideal model system for functional studies [25, 26]. Our analysis identified 131 proteins in proximity to PsbO, including a previously uncharacterized hypothetical protein Ssl2814. These proteins could interact directly or indirectly with PsbO and regulate the PSII activity in light‐driven water splitting and the generation of electrons.

In this study, we demonstrate that Ssl2814 physically interacts with PsbO. Functional investigations further suggest that Ssl2814 plays a crucial role in coordinating nitrate assimilation and photosynthetic electron production. Based on these findings, we propose naming this protein PsbO‐interacting regulator of nitrate assimilation (PirN).

## 2 Results

### 2.1 PirN Interacts with PsbO

**FIGURE 1:**
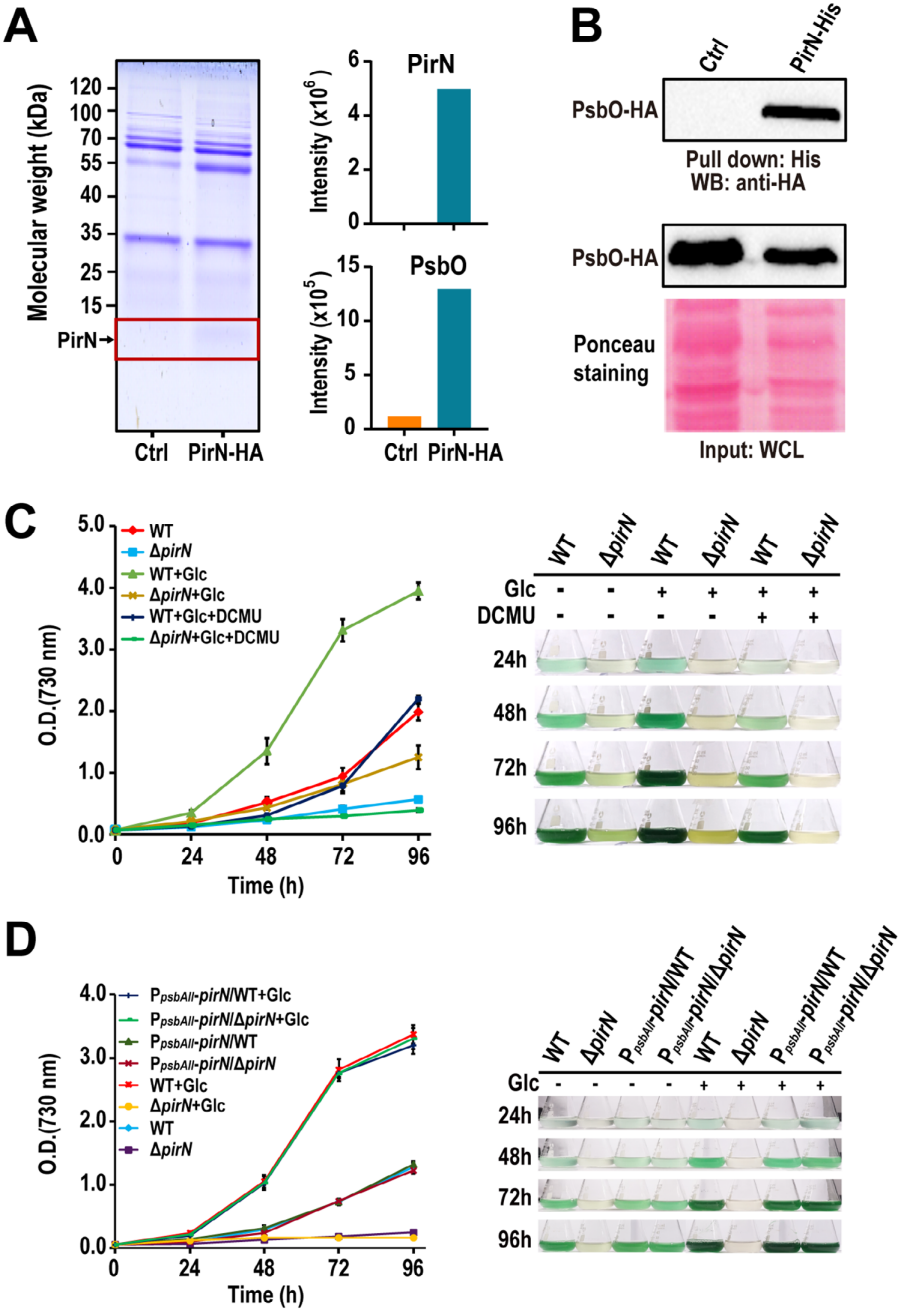
PirN protein interacts with PsbO and is essential for trophic growth of *Synechocystis*. (A) Whole cell lysates (WCL) from the WT (control: Ctrl) and the PirN‐HA knock‐in strain (PirN‐HA) of *Synechocystis* were subjected to immunoprecipitation (IP) using an anti‐HA antibody. The immunoprecipitated proteins were separated by SDS‐PAGE (left panel), followed by in‐gel digestion and quantitative identification by mass spectrometry (MS) (right panel). (B) Whole‐cell lysates from the PsbO‐HA knock‐in strain of *Synechocystis* were incubated with Ni Sepharose resin charged with recombinant His‐tagged PirN (PirN‐His) or with uncharged resin as a control (Ctrl). Proteins pulled down by PirN‐His were probed for PsbO‐HA using an anti‐HA antibody. Input samples were also probed for PsbO‐HA, which served as a loading control. (C,D) Growth curves of the WT and Δ*pirN* (C) and corresponding PirN‐complemented *Synechocystis* strains (D) cultured under the indicated conditions (left panel). Data are presented as the mean ± SD from three biological replicates (*n* = 3). The cultures were photographed every 24 h to monitor cell density and color changes (right panel). Glc: glucose.

The proximity of PirN to PsbO suggests a potential interaction between the two proteins [24], and this was confirmed by immunoprecipitation coupled with mass spectrometry (IP‐MS) analysis. PirN, fused with a C‐terminal HA tag, was ectopically expressed in the wild type (WT) background. The whole cell lysates of the strain expressing PirN‐HA and the WT (control) were subjected to immunoprecipitation with an anti‐HA antibody. Co‐immunoprecipitated proteins were separated by sodium dodecyl sulfate‐polyacrylamide gel electrophoresis (SDS‐PAGE), in‐gel digested, and identified via MS. The results revealed a significantly higher amount of co‐immunoprecipitated PsbO in the strain expressing PirN‐HA compared to the control, indicating an interaction between PirN and PsbO (Figure 1A; Table S1). To further confirm this interaction, recombinant PirN with a C‐terminal 6x His tag was purified from *E. coli* and incubated with the lysates from a *Synechocystis* strain expressing a PsbO‐HA‐APEX fusion protein [24]. Western blot analysis of the pull‐down proteins showed that PsbO was specifically pulled down by PirN (Figure 1B). These findings provide strong evidence supporting a specific interaction between PirN and PsbO. Notably, PII protein, a global regulator of nitrogen assimilation [23], was also identified and validated as a PirN‐interacting partner using the similar IP‐MS and Western‐blot approaches (Figure S1 and Table S1).

### 2.2 PirN Plays a Pivotal Role in the Growth of *Synechocystis* Under Nitrate Conditions

Phylogenetic analyses indicate that PirN homologs are predominantly conserved in freshwater and terrestrial cyanobacterial lineages, including members of the *Nostocales* and *Oscillatoriales*, as well as in some cyanobacteria inhabiting both freshwater and coastal environments (Figure S2A). In contrast, PirN is absent from dominant marine cyanobacteria, such as *Prochlorococcus* spp. and most marine *Synechococcus* ecotypes. This absence may reflect adaptation to nutrient‐poor oceanic environments, where nitrate is not the primary nitrogen source. (Figure S2A). All homologs in the cyanobacteria contain DUF1830 (Figure S2B), a domain with unknown function that is also present in the protein PirC/CfrA, a protein involved in the regulation of nitrogen and carbon assimilation [22, 27]. To investigate the function of PirN, a knockout mutant was generated for the open reading frame (ORF) *pirN* through homologous recombination as previously described (Figure S3A) [28, 29], and the complete segregation of the mutant was confirmed by PCR (Figure S3B). To investigate whether the deletion of *pirN* impacts the expression of the ORFs localized in the same genomic place with *pirN*, a polar effect that usually occurs in prokaryotes [26, 28], we examined the transcription of the ORFs *sll1450*, *sll1451*, *sll1452*, and *sll1453* in the upstream and *sll1454*, *sll1455*, *slr1552*, and *sll1456* in the downstream of *pirN* by using RT‐PCR (Figure S3C). The results showed that the transcription of *pirN* was undetectable in Δ*pirN* mutant relative to the WT, whereas no significant difference in transcription was detected for the other ORFs (Figure S3D,E). Thus, a *pirN*‐deletion mutant (Δ*pirN*) was successfully generated without significantly altering the expression of its neighboring ORFs.

To investigate the growth phenotype of Δ*pirN* mutant, we compared the growth curves of the WT and the mutant strains in BG‐11 medium with nitrate as the sole nitrogen source under photoautotrophic, photoheterotrophic, and photomixotrophic conditions. The growth of Δ*pirN* mutant was significantly affected under all tested conditions compared to the WT (Figure 1C). To confirm that the growth defect was caused by the depletion of PirN, we generated a complement strain (P*_psbAII_*‐*pirN*/Δ*pirN*) ectopically expressing PirN under the control of the *psbAII* promoter, integrated at a neutral genomic locus where the ORF *slr0168* is located [30]. Complete segregation and PirN expression in the complemented strain were confirmed by PCR analysis and Western blotting (Figure S4). As expected, the growth inhibition of Δ*pirN* mutant was almost completely rescued by the ectopic expression of PirN, whereas the P*_psbAII_*‐*pirN*/WT strain showed negligible growth difference compared to the WT (Figure 1D). Collectively, these results suggest that PirN plays a pivotal role in the growth of *Synechocystis*.

### 2.3 PirN Regulates PsbO Expression and Photosynthetic Activities

**FIGURE 2:**
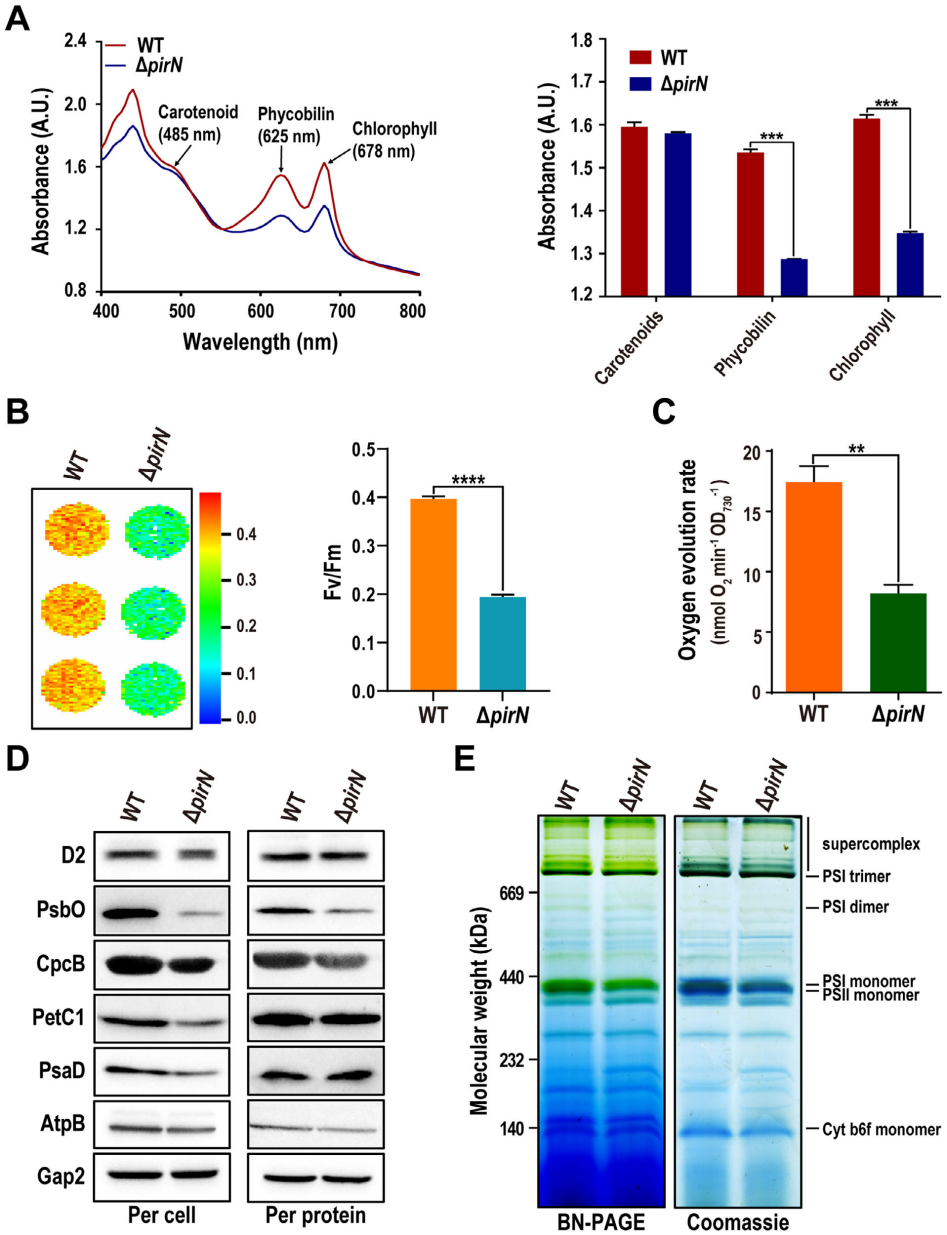
PirN depletion reduced PSII level. (A) Representative whole cell absorption spectrum (400–800 nm) of the WT and Δ*pirN*. Spectra were normalized to an optical density of 1.0 at 730 nm. Absorption peaks corresponding to chlorophyll, phycobilins, and carotenoids at the indicated wavelengths are labeled (left panel) and quantified (right panel). Data are presented as the mean ± SD from three biological replicates (*n* = 3). The statistical significance was determined by Student's t‐test. ^***^: *p* < 0.001. (B) Chl fluorescence imaging of the indicated strains showing Fv/Fm values (left panel), with quantitative results shown in the bar graph (right panel). Data are presented as the mean ± SD of three biological replicates (*n* = 3). Statistical significance was assessed via Student's t‐test. ^****^: *p* < 0.0001. (C) The bar graph shows the oxygen evolution rate of the indicated strains. Data are presented as the mean ± SD from three biological replicates (*n* = 3). Student's t‐test was used for statistical analysis. ^**^: *p* < 0.01. (D) Western blot detection of representative photosynthesis‐related proteins in the indicated strains. Blots were performed under two loading conditions: equal cell number (per cell) and equal total protein amount (per protein). Gap2 was used as the loading control in this and all subsequent Western blot experiments. (E) Separation and visualization of thylakoid membrane protein complexes from the indicated strains using Blue native (BN)‐PAGE (left panel). The gel was subsequently stained with Coomassie Brilliant Blue (CBB). The experiment was conducted on a per‐cell basis. Known protein complexes are labeled on the right.

Unlike the blue–green WT, Δ*pirN* mutant displays a pale green or yellowish hue under all tested conditions (Figure 1C), indicating a reduction in chlorophyll and phycobilin content, both crucial for photon absorption [31]. Whole‐cell absorbance measurements confirmed significantly lower absorbance in the mutant at 678 and 625 nm, corresponding to chlorophyll and phycobilin, respectively. In contrast, absorbance at 485 nm, associated with carotenoids, showed minimal difference between the mutant and WT (Figure 2A). Notably, the intensity of the peak at approximately 430 nm was also significantly reduced in the Δ*pirN* mutant (Figure 2A). This peak corresponds to the Soret absorption band of chlorophyll a, which enables chlorophyll a to absorb high‐energy blue light [32]. The reduced intensity of this peak in the Δ*pirN* mutant likely reflects decreased chlorophyll a content and/or alterations in photosystem structure or function. Chlorophyll fluorescence measurements further revealed a marked decrease in the maximum quantum efficiency of PSII photochemistry (Fv/Fm) in Δ*pirN* mutant compared to the WT (Figure 2B). Consistently, the mutant exhibited a significantly reduced oxygen evolution rate (OER) (Figure 2C). These findings collectively indicate that the Δ*pirN* mutant suffers from impaired photosynthetic activity.

To further assess the impact of PirN depletion on photosynthesis, we measured the levels of key protein components from major photosynthetic complexes using Western blotting. Measurements were performed based on an equal number of cells and equal amounts of extracted proteins. In both cases, PsbO, CpcB, and AtpB were significantly downregulated in Δ*pirN* mutant (Figure 2D; Figure S5). Potential changes in the major photosynthetic complexes within the thylakoid membrane were further analyzed using Blue Native polyacrylamide gel electrophoresis (BN‐PAGE）, revealing a reduction in PSI and PSII monomer levels in the mutant (Figure 2E). Together, these findings indicate that PirN depletion leads to the downregulation of specific photosynthetic proteins and disrupts the accumulation of key photosynthetic macrocomplexes. Notably, a band of approximately 200 kDa shows a marked increase in intensity in the Δ*pirN* mutant compared with the WT (Figure 2E). Mass spectrometry analysis revealed that this band is highly enriched in proteins involved in nitrogen transport and assimilation, including ammonium, nitrate, and urea transporters, as well as glutamate–ammonia ligase (GlnN) (Table S2). These results suggest that nitrogen‐related transporters and GlnN may assemble into a macrocomplex that is upregulated in the mutant.

**FIGURE 3:**
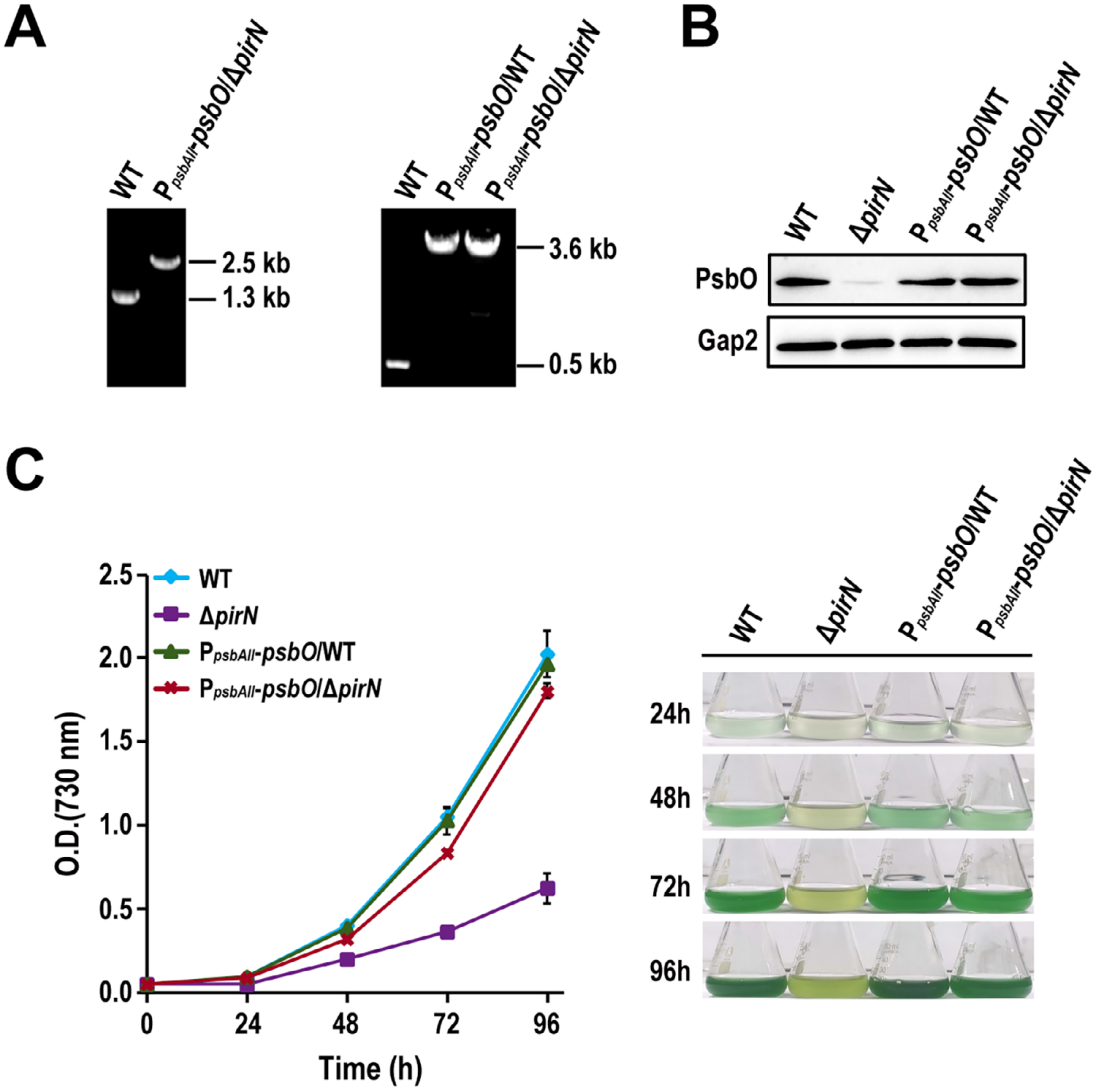
PsbO complementation largely rescues the growth defect of the Δ*pirN* mutant. (A) PCR confirmation of complete segregation of the indicated mutant strains. Primers for amplifying *pirN* (left panel) and *slr0168* (right panel) were used. (B) Western blot detection of PsbO in the indicated strains. (C) Growth curves of the indicated strains under photoautotrophic conditions (left panel). Data are presented as the mean ± SD from three biological replicates (*n* = 3). The cultures were photographed every 24 h to monitor cell density and color changes (right panel).

The interaction between PirN and PsbO suggests that the significant downregulation of PsbO may contribute to the growth defect observed in Δ*pirN* mutant (Figure 1). To test this hypothesis, we attempted to restore PsbO levels in the mutant by ectopically expressing PsbO‐HA under the control of the *psbAII* promoter in the Δ*pirN* background (P*_psbAII_*‐*psbO*/Δ*pirN*). PCR confirmed successful segregation of the mutant (Figure 3A), and Western blot analysis validated the expression of the PsbO fusion protein, showing that PsbO levels in P*_psbAII_*‐*psbO*/Δ*pirN* mutant were comparable to those in the WT (Figure 3B). Growth experiments showed that the growth of P*_psbAII_*‐*psbO*/Δ*pirN* mutant was largely, though not fully, restored compared to the WT (Figure 3C), suggesting that PsbO downregulation is the primary contributor to the growth defect observed in Δ*pirN* mutant.

Additionally, we generated a P*_psbAII_*‐*psbO*/WT strain, which ectopically expresses PsbO‐HA in the WT background as a control. Notably, PsbO levels were comparable between P*_psbAII_*‐*psbO*/WT and P*_psbAII_*‐*psbO*/Δ*pirN* mutants (Figure 3), despite the presence of the endogenous *psbO* in the former. This result suggests the existence of an unknown regulatory mechanism that prevents overaccumulation of PsbO in *Synechocystis*, which is essential for maintaining proper stoichiometry and structural integrity of the oxygen‐evolving complex.

### 2.4 PirN Plays a Critical Role in Regulating Nitrate Assimilation

**FIGURE 4:**
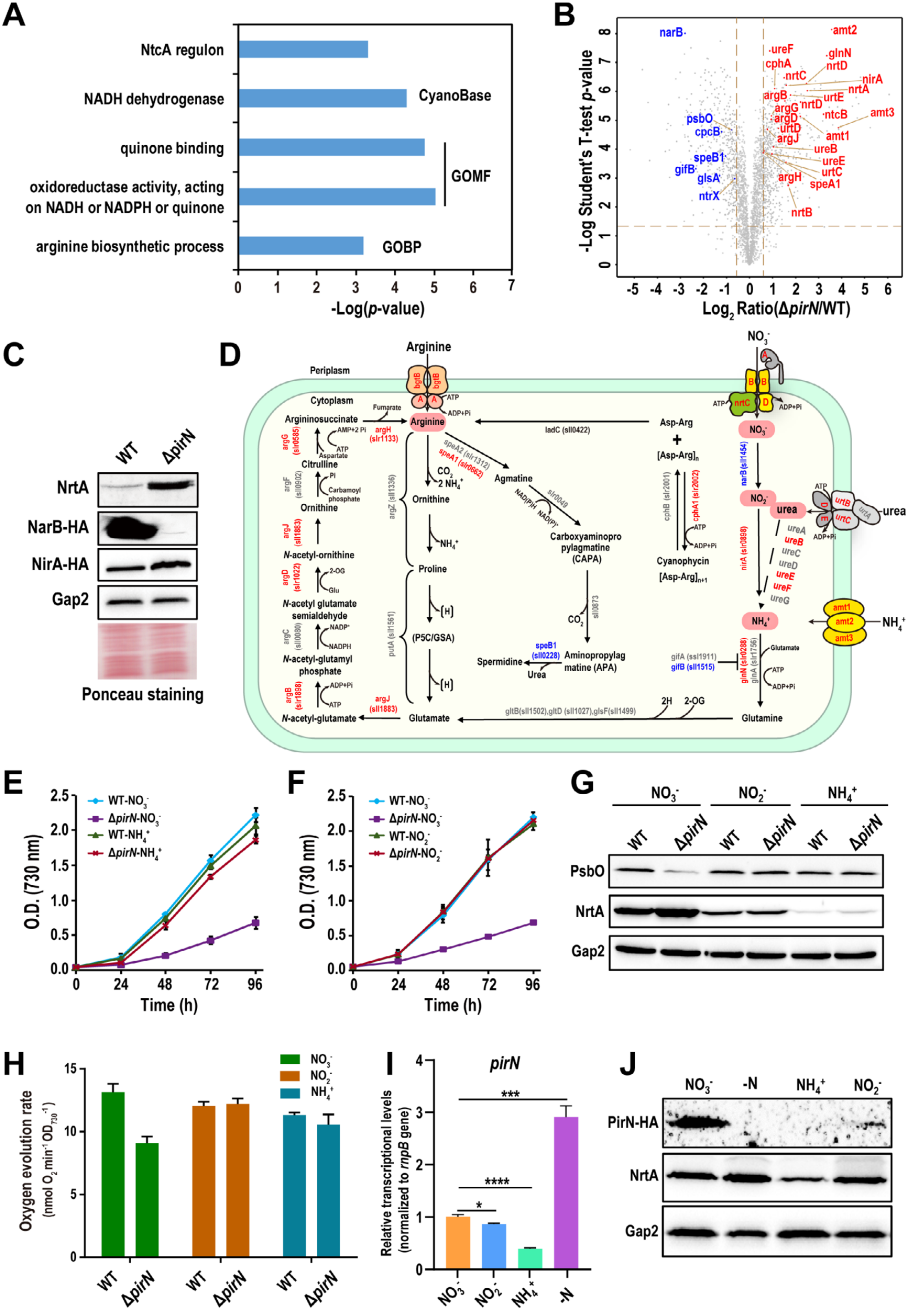
PirN plays a critical regulatory role specifically in nitrate assimilation. (A) Bar graph showing enriched functional categories among upregulated proteins in Δ*pirN* mutant. Functional enrichment analysis was performed using Fisher's exact test. Enriched terms include Gene Ontology terms for molecular function (GOMF) and biological process (GOBP), CyanoBase functional classifications, and members of the NtcA regulon. (B) Volcano plot showing differentially expressed proteins (DEPs) in Δ*pirN*. PsbO and proteins involved in nitrogen assimilation and metabolism were labeled. The dashed lines indicate the thresholds for fold change (vertical) and *p*‐value (horizontal). (C) Western blot validation of differential expression of the indicated proteins. Probed proteins include either endogenous proteins or HA‐tagged knock‐in variants in WT and Δ*pirN* backgrounds. Ponceau S staining was used as a loading control. (D) Schematic representation of the nitrogen assimilation and metabolism network in cyanobacteria. Proteins that are upregulated, downregulated, or unchanged in the Δ*pirN* mutant are indicated in red, blue, and gray, respectively. (E,F) Growth curves of WT and Δ*pirN* strains cultured with ammonium (E) or nitrite (F) as the nitrogen source. Cultures grown with nitrate were included as controls. Data are presented as the mean ± SD from three biological replicates (*n* = 3). (G) Western blot analysis of the indicated proteins in WT and Δ*pirN* strains grown under the specified nitrogen sources. Gap2 was used as an internal loading control. (H) Bar graph showing oxygen evolution rates of WT and *pirN* strains grown under the indicated nitrogen sources. Data are presented as the mean ± SD from three biological replicates (*n* = 3). (I) qPCR analysis of *pirN* transcript abundance in *Synechocystis* cultures grown with specified nitrogen sources or under nitrogen starvation (−N). The *rnpB* gene was used as an internal control. Transcript levels of each gene were normalized to the corresponding WT value. Data represent the mean ± SD of three biological replicates (*n* = 3). Statistical significance was determined using Student's t‐test. ^*^: *p* < 0.05, ^***^: *p* < 0.001, ^****^: *p* < 0.0001. (J) Western blot analysis of the indicated proteins in *Synechocystis* grown under the specified nitrogen sources. −N indicates a nitrogen starvation condition.

To investigate the mechanism underlying the growth defect in Δ*pirN*, we identified differentially expressed proteins (DEPs) using a data‐independent acquisition (DIA)‐based quantitative proteomics approach [33, 34, 35] (Figure S6). DEPs were determined using thresholds of *p* < 0.05 and a fold change >1.5 [36]. Among the 2808 proteins identified, 639 were upregulated, and 426 were downregulated in Δ*pirN* mutant, respectively (Table S3). Functional enrichment assay reveals that proteins associated with PSII and phycobilisome are enriched among the downregulated proteins (Table S4), consistent with the chlorophyll fluorescence and Western blotting results (Figure 2). In contrast, functions related to oxidoreductase activity, quinone binding, arginine biosynthetic process, and NtcA regulon are enriched among the upregulated proteins (Figure 4A). Notably, proteins within the NtcA regulon are primarily involved in nitrogen assimilation and metabolism [37]. This observation suggests that the nitrogen assimilation and cellular redox status balance may be disrupted in Δ*pirN* mutant. Indeed, the majority of proteins critical for nitrogen assimilation were upregulated in the mutant, including nitrate/nitrite transporters (NrtABCD), ammonium permeases (Amt1‐3), urea transporters (UrtD, E), NirA, and the glutamine synthetases GlnN, whereas the glutamine synthetase inhibitor GifB, a protein that negatively regulates nitrogen assimilation [38], was significantly downregulated (Figure 4B). Remarkably, NarB, the protein critical for nitrate assimilation, was significantly downregulated (Figure 4B). The differential expression of NrtA, NarB, and NirA was further confirmed by Western blotting (Figure 4C), and DEPs were mapped to a network for nitrogen assimilation and metabolism (Figure 4D).

The altered expression pattern of these proteins, except for NarB, together with the observed growth phenotype, mimicked a nitrogen starvation response [8, 9, 39, 40], despite sufficient nitrate being available in the growth medium. These findings suggest that PirN depletion disrupts nitrate assimilation, triggering a nitrogen starvation‐like state. Supplementing the Δ*pirN* mutant with ammonium or nitrite largely rescued the growth defect seen under nitrate conditions (Figure 4E,F; Figure S7), and the mutant exhibited no significant difference in PsbO expression with the WT under these conditions (Figure 4G). These results, along with the downregulation of NarB, indicate that the conversion of nitrate to nitrite, a crucial step in nitrate assimilation, was severely impaired in the mutant, leading to nitrogen starvation‐like symptoms.

In cyanobacteria, nitrate assimilation involves two key steps: reduction of nitrate to nitrite by NarB, followed by the reduction of nitrite to ammonium by NirA [41]. Both reactions rely on electrons provided by photoreduced Fd [42]. Existing evidence suggests that nitrate enhances electron transport from water to Fd by increasing the OER [43]. To assess whether oxygen evolution was affected in the mutant, we measured the OER in cells cultured with nitrate, nitrite, or ammonium (Figure 4H). Compared to the WT, Δ*pirN* mutant showed a significantly reduced OER when grown with nitrate but exhibited no significant difference under nitrite or ammonium conditions. Notably, in the WT, the highest OER was observed under nitrate conditions, while the lowest occurred with ammonium (Figure 4H), consistent with the electron availability provided by the oxygen‐evolving process.

To further explore the role of PirN in nitrate assimilation, we examined both *pirN* mRNA and protein levels in WT *Synechocystis* cells grown with nitrate, nitrite, ammonium, or under nitrogen‐starvation conditions, using qPCR and Western blot analysis, respectively. qPCR analysis showed that *pirN* mRNA levels under nitrogen starvation were approximately two‐fold higher than those under nitrate or nitrite conditions. The lowest *pirN* mRNA level was detected in ammonium‐grown cells, where it was less than 50% of that observed under nitrate conditions (Figure 4I). Given that *pirN* is located within an NtcA‐regulated operon containing several nitrogen assimilation‐related genes (Figure S3C) [41], this transcriptional pattern is consistent with previous observations that nitrogen assimilation genes are upregulated during nitrogen starvation and downregulated upon ammonium supplementation [8, 37].

Intriguingly, Western blot analysis revealed that PirN protein abundance does not correlate with *pirN* mRNA levels across these growth conditions. Specifically, PirN accumulated to a substantial level in nitrate‐grown cells but was barely detectable under the other conditions (Figure 4J), particularly during nitrogen starvation, in which *pirN* mRNA levels were highest. Together, these results indicate that transcriptional regulation of *pirN* does not coincide with its translational or post‐translational regulation, and that PirN functions specifically in nitrate assimilation, likely by coordinating the protein abundance of NarB and PsbO (Figure 4B), while being dispensable for nitrite or ammonium assimilation.

### 2.5 NarB Downregulation Partially Contributes to the Growth Defect of Δ*pirN* Mutant

**FIGURE 5:**
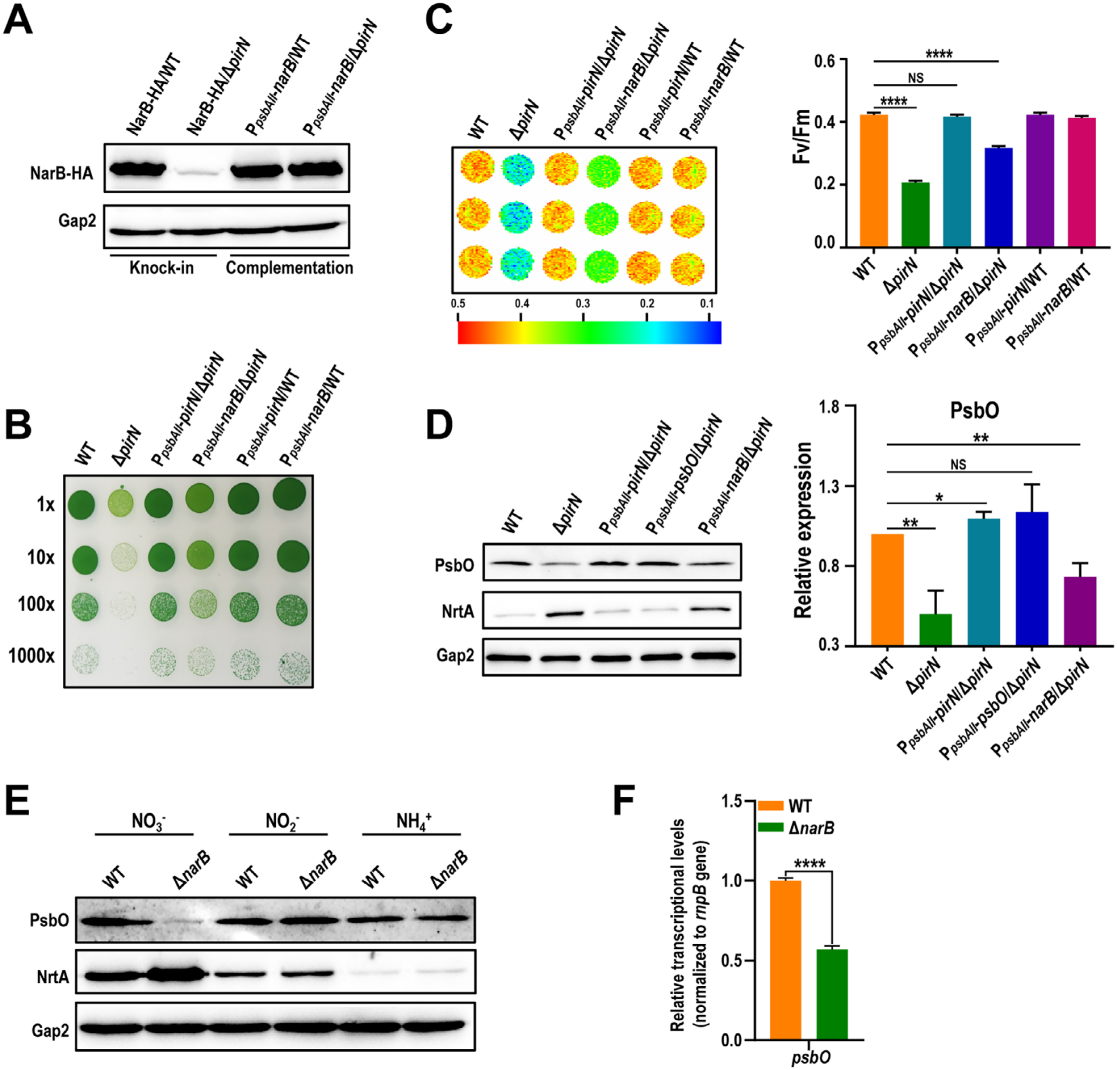
NarB complementation partially rescues the growth defect of Δ*pirN*. (A) Western blot detection of NarB‐HA in the indicated NarB knock‐in and NarB‐complementation strains. (B) Growth of the indicated strains on solid BG‐11 medium. Liquid cultures were serially diluted to an OD_730_ of 0.1 (1×), 0.01 (10×), 0.001 (100×), and 0.0001 (1000×), and 6 µL of each dilution was spotted onto agar plates. Plates were incubated under photoautotrophic conditions for 7 days. (C) Chlorophyll fluorescence imaging of the indicated strains showing Fv/Fm values (left panel), with quantitative results shown in the bar graph (right panel). Data represent the mean ± SD of three biological replicates (*n* = 3). Statistical significance was evaluated by Student's t‐test. NS: not significant, ^****^: *p* < 0.0001. (D) Western blot detection of PsbO and NrtA in the indicated strains (left panel). PsbO levels in each mutant were quantified relative to those in the WT using ImageJ software (right panel). Data represent the mean ± SD of three biological replicates (*n* = 3). Statistical significance was determined using Student's t‐test. NS: not significant, ^*^: *p* < 0.05, ^**^: *p* < 0.01. (E) Western blot detection of PsbO and NrtA in the WT and Δ*narB* strains cultured with indicated nitrogen sources. (F) Bar graph showing the relative mRNA levels of *psbO* in the indicated strains, measured by qPCR. The *rnpB* gene was used as an internal control. Transcript levels of each gene were normalized to the corresponding WT value. Data represent the mean ± SD of three biological replicates (*n* = 3). Statistical analyses were performed using Student's t‐test. ^****^: *p* < 0.0001.

Existing evidence suggests that NarB inactivation impairs the conversion of nitrate to nitrite and disrupts overall nitrogen assimilation [41]. To determine the extent of the contribution to the impaired growth of Δ*pirN* mutant by NarB downregulation, we ectopically expressed NarB under the control of the *psbAII* promoter in the Δ*pirN* mutant background (P*_psbAII_*‐*narB*/Δ*pirN*). Successful construction of the mutant was confirmed by PCR, and NarB expression was confirmed by Western blotting (Figure S8). The ectopic expression restored NarB to WT levels (Figure 5A), which was essential for assessing its role in the Δ*pirN* mutant phenotype.

Growth assays revealed that NarB complementation only partially rescued the growth defect of Δ*pirN* mutant. In contrast, PirN complementation fully rescued the growth defect (Figure 5B; Figure S9). Consistently, the maximum photochemical efficiency of PSII was partially recovered by NarB complementation but fully recovered by PirN complementation (Figure 5C). These findings suggest that although NarB downregulation contributes to the Δ*pirN* mutant phenotype, restoring NarB to WT levels is insufficient to fully restore photosynthetic activity, which likely accounts for the partial recovery in growth.

Given that PsbO complementation was more effective than NarB complementation in rescuing the growth defect of Δ*pirN* mutant (Figure 3), we examined PsbO levels in P*_psbAII_*‐*narB*/Δ*pirN* by Western blotting. As expected, PsbO level was elevated in the mutant but remained below that of the WT (Figure 5D), consistent with the observed partial recovery of growth and photosynthetic activity (Figure 5B,C). Notably, while complementation of either PirN or PsbO in Δ*pirN* mutant successfully reduced NrtA expression to the WT level, NarB complementation did not significantly suppress NrtA expression (Figure 5D). This result indicates that NarB complementation alone is insufficient to fully compensate for the effects of PirN deletion.

The downregulation of both NarB and PsbO in Δ*pirN* mutant, along with the partial restoration of PsbO expression by NarB complementation (Figures 4B and 5D), suggests that NarB may play a role in regulating PsbO expression. Probing PsbO in Δ*narB* mutant grown under nitrate, nitrite, and ammonium conditions by Western‐blotting indicates that PsbO was significantly downregulated in Δ*narB* mutant grown with nitrate, but not with nitrite or ammonium (Figure 5E). Consistently, the transcript level of *psbO* was also significantly downregulated in the Δ*narB* mutant grown with nitrate (Figure 5F). This expression pattern closely mirrors that observed in Δ*pirN* mutant under different nitrogen sources (Figure 4G). These findings, together with the partial recovery of PsbO in Δ*pirN* mutant upon NarB complementation (Figure 5D), suggest that NarB is necessary but not sufficient for PsbO expression specifically under nitrate conditions.

### 2.6 PirN and PsbO Regulate NarB Expression at the Post‐Transcriptional Level

**FIGURE 6:**
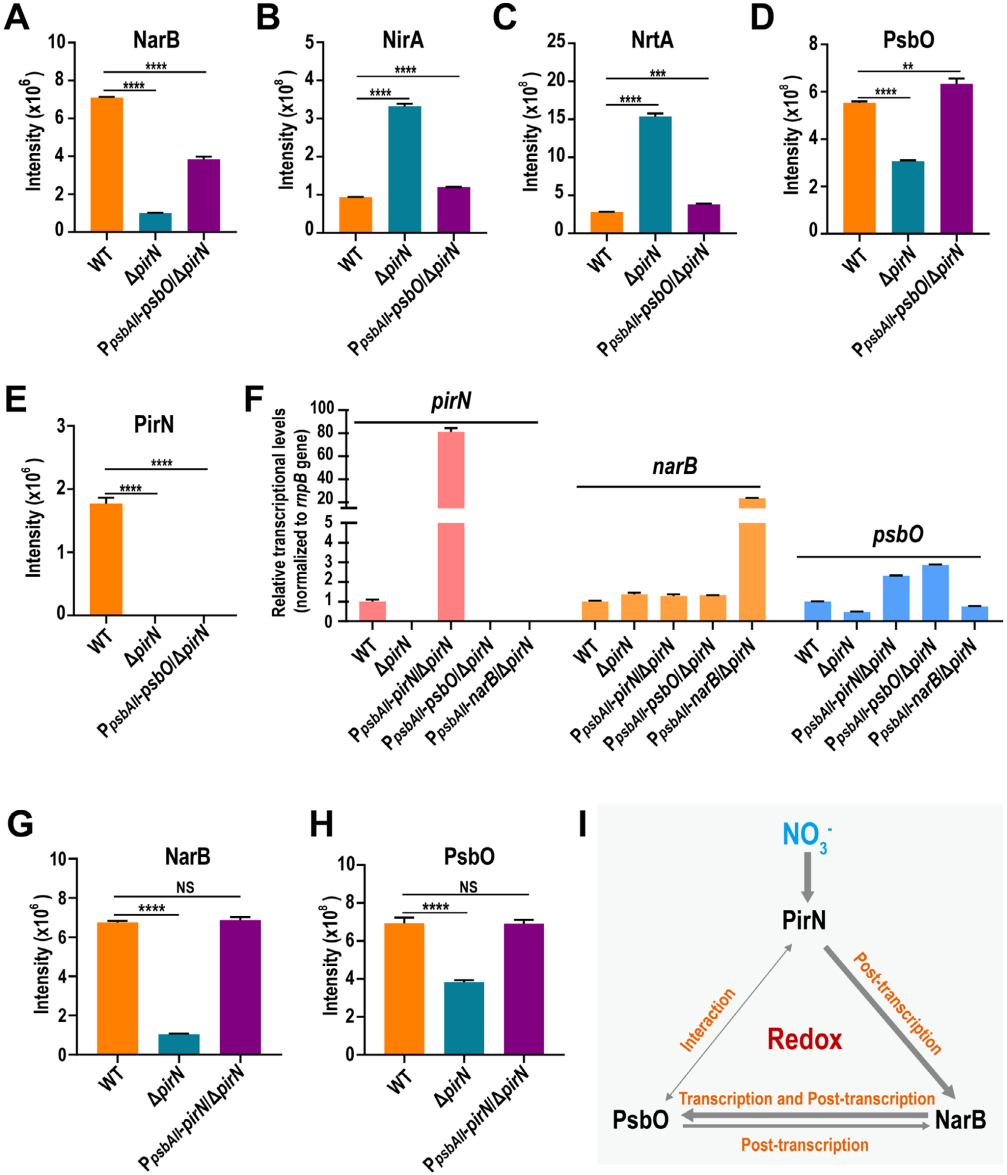
Analysis of the regulatory relationships among PirN, NarB, and PsbO at both the mRNA and protein levels. (A–E) Bar graphs showing the relative protein abundances of NarB (A), NirA (B), NrtA (C), PsbO (D), and PirN (E) in the indicated *Synechocystis* strains, quantified by a DIA‐based quantitative approach. Data represent the mean ± SD of three biological replicates (*n* = 3). Student's t‐test was used for statistical analysis. ^****^: *p* < 0.0001, ^***^: *p* < 0.001, ^**^: *p* < 0.01. (F) Bar graph showing the relative mRNA levels of *pirN*, *narB*, and *psbO* in the indicated strains, measured by qPCR. The *rnpB* gene was used as an internal control. Transcript levels of each gene were normalized to the corresponding WT value. Data represent the mean ± SD of three biological replicates (*n* = 3). (G,H) Bar graphs showing the relative protein abundances of NarB (G), and PsbO (H) in the indicated *Synechocystis* strains, quantified by a DIA‐based quantitative approach. Data represent the mean ± SD of three biological replicates (*n* = 3). Student's t‐test was used for statistical analysis. NS: not significant, ^****^: *p* < 0.0001. (I) Schematic illustration of the regulatory relationships among PirN, NarB, and PsbO in cyanobacteria under nitrate conditions.

The rescue of the Δ*pirN* mutant growth defect by restoring PsbO to WT levels suggests that NarB expression may also be elevated in the PsbO‐complemented strain (Figure 3). To explore this, we performed quantitative proteomic analysis of WT, Δ*pirN*, and P*_psbAII_*‐*psbO*/Δ*pirN* mutant strains (Table S5). The results showed that NarB expression in P*_psbAII_*‐*psbO*/Δ*pirN* was significantly elevated compared to Δ*pirN* mutant, but is still below that of the WT (Figure 6A; Figure S10). Consistently, the elevated levels of NirA and NrtA observed in Δ*pirN* were restored to WT levels upon PsbO complementation (Figures 4B,C and 6B–E). Given that NarB complementation did not effectively suppress NrtA upregulation (Figure 5D), these results indicate that PsbO complementation provides a more comprehensive rescuing effect than NarB complementation in the Δ*pirN* mutant.

To further investigate the regulatory relationships among *pirN*, *psbO*, and *narB*, we measured their transcript levels in WT and related mutants using qPCR (Figure 6F). The results indicate that in Δ*pirN* mutant while *narB* transcription was slightly increased (Figure 6F; Figure S3), *psbO* transcription was suppressed to 40%, underscoring the importance of *pirN* for *psbO* expression, particularly at the transcriptional level. Remarkably, although PsbO complementation partially recovered NarB in Δ*pirN* mutant (Figure 6A), this recovery likely occurred post‐transcriptionally, as *narB* transcription did not significantly differ between Δ*pirN* and P*_psbAII_*‐*psbO*/Δ*pirN* mutants and is comparable to that of the WT (Figure 6F). The poor correlation between *narB* mRNA and protein levels among the three *Synechocystis* strains may be attributable to differences in translation efficiency and/or protein stability, the latter potentially being influenced by differential post‐translational modifications, including redox modifications of cysteine residues. In contrast, NarB complementation only partially recovered *psbO* at both protein and mRNA levels in Δ*pirN* mutant (Figures 5D and 6F). Notably, complementation of *pirN* in the Δ*pirN* mutant background fully restored NarB and PsbO protein levels (Figure 6G,H), further underscoring the essential role of PirN in regulating NarB and PsbO, which function in nitrogen assimilation and photosynthesis, respectively, in cyanobacteria.

Based on these results, we can clearly delineate the regulatory relationships among PirN, NarB, and PsbO (Figure 6I). In the presence of nitrate, PirN is expressed and accumulates to a sufficient level (Figure 4J), playing a key role in the post‐transcriptional regulation of NarB (Figures 4B,C and 6A,F). NarB, in turn, regulates both *psbO* mRNA and PsbO protein abundance (Figure 5E,F). Conversely, PsbO also influences the protein and mRNA levels of NarB post‐transcriptionally, although to a lesser extent (Figure 6A,F). Given that PsbO and NarB are localized in distinct and physically separated cellular compartments, their regulatory interaction is likely indirect, potentially mediated by changes in the cellular redox state. In addition, PirN and PsbO directly interact, though the functional significance of this interaction remains to be elucidated. It is important to note that these regulatory relationships are specific to nitrate conditions. Under ammonium or nitrite conditions, PirN is either not expressed or is significantly downregulated, and the expressions of NarB and PsbO appear to be independent of each other (Figures 4G,J and 5E).

### 2.7 PirN Couples Nitrate Assimilation to Photosynthesis

**FIGURE 7:**
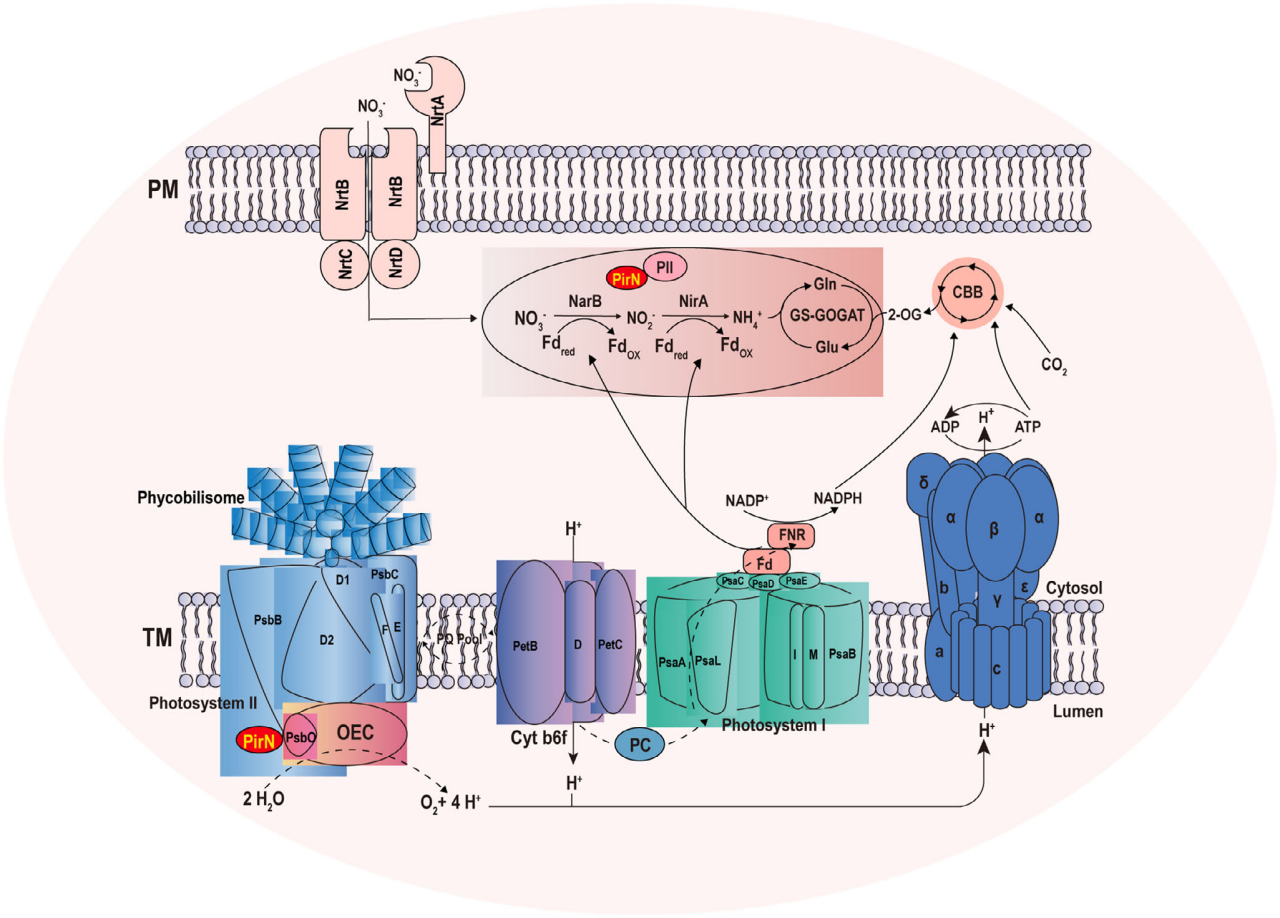
A proposed working model illustrating how PirN coordinates nitrate assimilation with photosynthetic electron transport. TM: thylakoid membrane. PM: plasma membrane. PC: plastocyanin.

Although photosynthetic electron production and nitrogen assimilation are biochemically linked [44], little is known about how these two processes are coordinately regulated. Based on the functional characterization of PirN (Figures 1, 2, 3, 4, 5, 6), we propose a model linking PSII activity to nitrate assimilation (Figure 7). In this model, PirN is specifically expressed under nitrate conditions (Figure 4J), with a fraction transported to the thylakoid lumen, where it interacts with PsbO to modulate PSII oxygen‐evolving activity and photosynthetic electron flow. Another fraction of PirN remains in the cytoplasm, where it interacts with other binding partners and regulates *narB* translation (Figure S1). The balanced distribution and different functions of PirN between the cytoplasm and the thylakoid lumen are therefore crucial for the coordinated regulation of photosynthesis and nitrate assimilation. Depletion of PirN severely impairs *narB* translation (Figures 4B,C and 6F), leading to defective nitrate assimilation. The resulting inability to dissipate excess photosynthetically generated electrons through nitrate assimilation causes severe redox stress, prompting the Δ*pirN* mutant to downregulate PsbO abundance and oxygen‐evolving activity (Figure 4G,H). Through PirN‐mediated coordinated regulation of NarB and PsbO, nitrate assimilation is thus coupled to PSII activity, helping to maintain cellular redox homeostasis. Nevertheless, a PirN‐independent coupling between these two processes also exists in nitrate‐grown cells, as evidenced by the partial restoration of NarB and PsbO levels upon overexpression of PsbO or NarB, respectively, in the Δ*pirN* background (Figures 5D and 6A). This reciprocal rescue strongly supports coordinated expression of the two proteins and, consequently, the coupling of nitrate assimilation with photosynthesis, reflecting an intrinsic cellular self‐rescue mechanism. Therefore, coupling nitrate assimilation with photosynthesis is essential both for optimal growth in the presence of PirN and for self‐rescue in its absence.

## 3 Discussion

PirN was previously identified as being in close proximity to PsbO [24], and contains a DUF1830 domain that is also present in the protein PirC/CfrA involved in nitrogen and carbon assimilation (Figure S2B) [22, 27]. These findings indicate that PirN may play roles in both photosynthesis and nitrogen assimilation. In this study, we confirmed that PirN directly interacts with PsbO and regulates its abundance in *Synechocystis* under nitrate‐grown conditions, but not when cultured with nitrite or ammonium (Figures 1A and 4G). PirN accumulates to a significant level in the presence of nitrate, whereas its abundance is markedly reduced or undetectable under nitrite or ammonium conditions (Figure 4J). PirN also regulates NarB abundance, likely at a translation or post‐translational level, as *narB* transcript levels show no notable difference between the Δ*pirN* mutant and the WT (Figure S3). The downregulation of NarB in Δ*pirN* leads to decreased PsbO abundance, supported by two independent lines of evidence: NarB complementation in Δ*pirN* mutant largely restores PsbO levels (Figure 5D), and deletion of *narB* alone results in PsbO downregulation comparable to that observed in Δ*pirN* mutant (Figures 4G and 5E). The downregulation of PsbO in both Δ*narB* and Δ*pirN* mutants likely reflects a broader regulatory redox response coordinating photosynthetic electron flow and nitrate assimilation. In both mutants, NarB is completely or largely depleted (Figure 4), leading to impaired nitrate assimilation. Because nitrate assimilation serves as an important sink for photosynthetically produced electrons, impairment of this process is expected to cause the accumulation of excess electrons and perturb intracellular redox homeostasis. Downregulation of PsbO and the consequent reduction in PSII activity would decrease electron production from water, thereby limiting excess electron accumulation and helping to maintain redox balance. In addition, the defective nitrate assimilation of the nitrate‐grown Δ*pirN* and Δ*narB* mutants may lead to severe carbon–nitrogen imbalance, necessitating downregulation of PsbO and, consequently, PSII activity, thereby preventing excess CO_2_ assimilation and maintaining optimal carbon–nitrogen balance in the two mutants. These findings indicate that PirN functions as a specific regulator that coordinates nitrate assimilation with photosynthetic electron transport. Given that nitrate is the most prevalent nitrogen source in many natural cyanobacterial habitats [13, 14], such regulation likely provides an adaptive advantage under fluctuating nitrate availability and light conditions, supporting cyanobacterial survival and proliferation.

The nitrate‐specific induction of PirN expression (Figure 4J), together with the regulatory connections among PirN, NarB, and PsbO (Figure 6I), provides important clues for uncovering a novel mechanism underlying the coordinated regulation of nitrate assimilation and photosynthesis. The negligible differences in *narB* mRNA levels between the WT and Δ*pirN* strains (Figure S3), together with the complete restoration of NarB protein abundance upon *pirN* complementation in the Δ*pirN* mutant background without a remarkable change in *narB* transcript levels (Figure 6F,G), strongly indicate that PirN does not regulate *narB* at the transcriptional level. When *narB* was ectopically expressed in Δ*pirN* mutant under the control of the *psbAII* promoter at a neutral genomic site distant from the native *narB* locus, transcript levels increased over 20‐fold relative to the WT (Figure 6F), yet its protein level remained comparable to that in the WT (Figure 5A). Together, these results suggest that PirN likely regulates NarB at the level of translation and that this regulation is critical (Figure 6I), as a >20‐fold increase in *narB* mRNA is required to achieve WT‐level NarB protein abundance in the absence of PirN (Figures 5A and 6F). We do not exclude the possibility that PirN may affect NarB protein stability in a redox‐sensitive way. However, due to the extremely low abundance of NarB in the absence of PirN (Figure 4C), it remains technically challenging to obtain definitive results from in vivo protein stability assays. The in vitro assay in which NarB‐HA was immunoprecipitated from a *Synechocystis* knock‐in strain expressing NarB‐HA was incubated with cell lysates from WT and Δ*pirN* strains revealed no obvious differences in degradation patterns. (Figure S11).

Significant downregulation of PsbO in both Δ*pirN* and Δ*narB* mutants and recovery in the corresponding complementing strains suggest that PirN‐regulated PsbO abundance depends on the presence of NarB, and the regulation probably occurs at the transcriptional level as indicated by the repressed transcription of *psbO* in Δ*pirN* (Figure 6F). In nitrate‐grown *Synechocystis*, the presence or absence of NarB determines whether excess electrons generated by PSII can be consumed through nitrate assimilation, thereby shaping cellular redox homeostasis and C/N balance. Defective nitrate assimilation resulting from complete or partial depletion of NarB, as observed in Δ*pirN* and Δ*narB* mutants (Figures 4B,C and 5E), is expected to trigger a signaling response that markedly reduces PsbO levels. This reduction is likely mediated through negative regulation of *psbO* transcription, translation, and/or protein stability (Figures 2D, 5E,F, and 6F), leading to reduced PsbO abundance and activity of the oxygen‐evolving complex and, consequently, decreased electron production (Figure 2). In this way, the Δ*pirN* mutant may alleviate redox stress and sustain slow but persistent growth under nitrate conditions.

The nitrate‐specific expression of PirN and its subsequent interactions with PsbO and other binding partners, such as PII may contribute to sensing photosynthetic electron flow. These interactions could facilitate signal transduction from the thylakoid lumen to the cytoplasm, potentially through differential associations of PirN with PsbO in the lumen and with its binding partners in the cytoplasm, ultimately regulating NarB abundance. Through this mechanism, PirN may enable nitrate‐specific coordinated regulation of PsbO and NarB, thereby linking photosynthetic electron production with nitrate assimilation (Figure 7).

Multiple layers of regulation involving various proteins have been uncovered in cyanobacteria to maintain intracellular C/N balance through coordinated nitrogen assimilation and carbon metabolism. Among these, the most extensively studied is the PII protein, which senses the intracellular level of the key carbon metabolite 2‐OG as well as the cellular energy status, and globally regulates the expression of proteins involved in nitrogen assimilation [23]. PirA, a PII‐interacting protein, competes with N‐acetylglutamate kinase (NAGK) for PII binding, thereby preventing NAGK activation and reducing nitrogen flux into arginine biosynthesis [45]. PirC, another PII‐interacting protein, binds, and inhibits 2,3‐phosphoglycerate‐independent phosphoglycerate mutase under nitrogen‐limited conditions, redirecting carbon flux toward glycogen synthesis [22]. More recently, NirP, encoded by a previously unannotated gene, was identified as a protein induced under low‐carbon conditions. NirP interacts with and inhibits nitrite reductase NirA, promoting nitrite excretion and contributing to the maintenance of C/N homeostasis [14]. In contrast to these regulators, PirN represents a distinct regulatory mechanism that acts further upstream, which is between photosynthetic electron transport and nitrate reduction, and highlights a previously uncharacterized layer of C/N balance control.

## 4 Experimental Section

### 4.1 Antibodies

The primary antibodies for Gap2, CpcB, NrtA, PsaD, and AtpB were provided by PhytoAB (San Jose, CA). The antibodies targeting PsbO, PetC1, and D2 were procured from Agrisera (Vännäs, Sweden). The monoclonal mouse antibody against His and HA was procured from CWBIO and MBL (Beijing, China), respectively.

### 4.2 Cell Culture

WT and mutant strains of *Synechocystis* were cultured at 30°C in BG‐11 medium containing 17.64 mm nitrate as the nitrogen source, unless otherwise specified. Cultures were grown under a photosynthetic photon flux density of 50 µmol m^−^^2^ s^−^^1^. For solid media, BG‐11 was supplemented with 1.5% (w/v) agar. Cells were harvested by centrifugation at 4000 × *g* for 10 min when cultures reached an optical density at 730 nm (OD_730_) of approximately 1.0, and used for biochemical and proteomic analyses. For experiments involving different trophic modes, cultures were supplemented as needed with 5 mm glucose or 5 mm 3‐(3,4‐dichlorophenyl)‐1,1‐dimethylurea (DCMU), while maintaining light illumination at 50 µmol m^−^^2^ s^−^^1^.

### 4.3 Mutant Generation

Gene knockout and knock‐in mutants were generated through homologous recombination using the procedures as we previously described [26, 28]. For the construction of overexpression strains, the coding sequences of the target genes were amplified from *Synechocystis* genomic DNA by PCR. The resulting amplicons were cloned into a pKW1188‐derived vector containing, in the following order, a 1500 bp DNA fragment immediately upstream of the open reading frame (ORF) *slr0168*, the *psbAII* promoter, an HA tag, a spectinomycin resistance cassette, and a 1320 bp internal fragment of the ORF *slr0168* (Figure S12). The cloning site is located between the *psbAII* promoter and the HA tag, into which an amplified target gene was inserted. Plasmid assembly was carried out using the pEASY‐Uni Seamless Cloning and Assembly Kit (TransGen Biotech, Beijing, China).

Final constructs were integrated into the chromosome of WT or mutant backgrounds at the neutral site corresponding to the ORF *slr0168* [29, 30]. Stable transformants were selected after multiple rounds of segregation on BG‐11 agar plates containing 20 µg/mL spectinomycin or kanamycin and were confirmed by PCR analysis. A complete list of primers and mutant strains used in this study is provided in Tables S6 and S7, respectively.

### 4.4 RNA Preparation, RT‐PCR, and RT‐qPCR

Total RNA was extracted from exponentially growing cells by the phenol–chloroform method, as previously described [46]. The isolated RNA was treated with RNase‐free DNase I (TransGen Biotech, Beijing, China) at 37°C for 1.5 h to eliminate residual genomic DNA (gDNA). To verify the absence of gDNA contamination, PCR analysis of the target genes was routinely performed for each DNase I‐treated RNA sample. Reverse transcription was carried out using the ThermoScript RT‐PCR System (Promega, Madison, WI). The resulting cDNA was used as a template for RT‐qPCR analysis using the CFX96 Touch Real‐Time PCR detection system (Bio‐Rad) or for semi‐quantitative RT‐PCR to assess gene expression in *Synechocystis*. The RNA subunit of the RNase P‐encoding gene *rnpB* was used as an internal control to normalize transcript levels of the target genes [47]. qPCR was performed using Taq Pro Universal SYBR qPCR Master Mix (Vazyme Biotech, Nanjing, China) with gene‐specific primers under a three‐step cycling protocol. The melting curve analysis was performed to confirm the amplification of single‐band DNA fragments in each reaction. Relative quantification of transcripts was conducted with the 2^(‐ΔΔCT)^ method [48]. The sequences of the forward and reverse PCR primer sets used in the RT‐PCR and RT‐qPCR are listed in Table S6. All PCR experiments were performed in triplicate.

### 4.5 Pigment Content Measurement

Absorption spectra of *Synechocystis* cultures were recorded in the 400–800 nm range using an Epoch microplate spectrophotometer (BioTek, USA). The absorbance peaks at 485, 625, and 678 nm were used to estimate the relative contents of carotenoids, phycobilins, and chlorophyll, respectively. To enable comparison across samples, all spectra were normalized to 1.0 of OD_730_.

### 4.6 Measurement of Chl Fluorescence and Oxygen Evolution Rate

Chl fluorescence was measured using a Fluorometer FluorCam 800 MF (Photon System Instruments, Brno, Czech Republic) as previously described [49]. Cell cultures were adjusted to an OD_730_ of approximately 1.0 and dark‐adapted for 15 min prior to measurement. A saturating light flash with a duration of 0.8 s was employed to determine Fm. The sensitivity and super were set at 20% and 60%, respectively, for the measurement.

The steady‐state oxygen evolution rate was measured using a Chlorolab‐2 oxygen electrode (Hansatech, Norfolk, UK) according to Spasic et al. [50]. WT and mutant *Synechocystis* strains were grown in liquid BG‐11 medium under various nitrogen sources until reaching the exponential growth phase (OD_730nm_ ∼ 1.0). Net photosynthesis was measured in the presence of 10 mm NaHCO_3_.

### 4.7 Blue Native Polyacrylamide Gel Electrophoresis (BN‐PAGE)

Total membrane fractions of *Synechocystis* were isolated as previously described [51]. Cell cultures were harvested and lysed in a SMN lysis buffer containing 0.4 mm sucrose, 50 mm 3‐(*N*‐morpholino) propanesulfonic acid (MOPS), pH 7.0, 10 mm NaCl, 5 mm EDTA, and 0.5 mm phenylmethanesulfonyl fluoride (PMSF) with a JXFS‐TRPR‐24L bead beater (Jingxin Technology, Shanghai, China). Insoluble debris was removed by centrifugation for 10 min at 5000 × *g* at 4°C. Membranes were pelleted by centrifugation at 100 000 × *g* at 4°C for 1 h.

BN‐PAGE of *Synechocystis* membranes was performed as previously described with slight modifications [52, 53, 54]. Isolated membranes were first washed with a buffer containing 330 mm sorbitol, 50 mm BisTris‐HCl (pH 7.0), 0.5 mm PMSF, then solubilized in 25 mm BisTris‐HCl (pH 7.0), 10 mm MgCl_2_, 20% glycerol, 0.1 U RNase‐free DNase, and 0.5 mm PMSF. After incubation on ice for 40 min with 2% *n*‐dodecyl β‐D‐maltoside, followed by centrifugation at 20 000 × *g* for 15 min at 4°C, the resulting supernatants were mixed with 1/10 volume of BN sample buffer (5% Serva Blue G, 100 mm BisTris‐HCl (pH 7.0), 30% sucrose, 500 mm ε‐amino‐*n*‐caproic acid, 10 mm EDTA). Solubilized membranes were then applied to a 5%–13% acrylamide gradient gel. Electrophoresis was carried out over 5 h with a stepwise increase in voltage from 50 to 200 V.

### 4.8 In‐Gel Digestion

Immunoprecipitated proteins were separated by SDS‐PAGE and visualized using Coomassie Brilliant Blue (CBB) staining. Each gel lane was cut into 2–3 mm^2^ slices in a dust‐free environment. The gel pieces were destained, reduced with 10 mm dithiothreitol (DTT) at 56°C for 1 h, and alkylated with 55 mm iodoacetamide at room temperature in the dark for 45 min. Proteins were digested in‐gel with trypsin at a 1:50 enzyme‐to‐substrate ratio at 37°C overnight. Peptides were extracted from the gel slices by sonication, dried using a Speed‐Vac concentrator, and redissolved in 0.1% formic acid (FA). The samples were then filtered through a 0.45 µm centrifugal filter prior to liquid chromatography–tandem mass spectrometry (LC‐MS/MS) analysis [55].

### 4.9 Pull‐Down Assay

Ni Sepharose 6 Fast Flow resin (Cytiva, USA) coated with recombinant His‐tagged bait protein was incubated with whole cell lysates containing potential prey proteins at 4°C overnight. Resin without bait protein was used as a negative control. After incubation, the beads were washed five times with 1× PBST (PBS with 0.1% Tween‐20), then resuspended in 3× SDS loading buffer and heated at 95°C for 10 min. Both input and pull‐down samples were analyzed by Western blotting.

### 4.10 Sample Preparation for Quantitative Proteomics

Protein samples were prepared according to Ge et al. [56]. Briefly, harvested cells were lysed in SMN buffer using a bead beater. The total cell extract was precipitated with ice‐cold 10% trichloroacetic acid in acetone at −20°C, washed with acetone, and resolubilized in 4% SDS in 0.1 m Tris‐HCl (pH 7.6). Protein concentration was determined using a BCA assay kit (Thermo Scientific, Rockford, IL, USA). Proteins were digested using the filter‐aided sample preparation method with slight modifications [26, 36]. Samples were reduced with 100 mm DTT and alkylated with 55 mm iodoacetamide. The denaturing buffer was exchanged with 0.1 m triethylammonium bicarbonate using Microcon YM‐30 centrifugal filter units (EMD Millipore, Billerica, MA). Proteins were digested with trypsin (Enzyme & Spectrum P01001, Beijing, China) at 37°C overnight. Tryptic peptides were then desalted using C18 StageTips [57], dried using a Speed‐Vac concentrator, and stored at −20°C. Before LC‐MS/MS analysis, peptides were resuspended in 0.1% FA.

### 4.11 LC‐MS/MS and Database Search

LC‐MS/MS analysis was performed using a Fusion Orbitrap Lumos mass spectrometer (Thermo Scientific, Rockford, IL, and Waltham, MA) coupled online to an Easy‐nLC 1000 system. Samples were analyzed in either DIA mode for quantitative proteomics or data‐dependent acquisition (DDA) mode for identification of immunoprecipitated proteins. Raw MS files were searched against the *Synechocystis* proteome database using MaxQuant [58] (version 1.6.3.4) for DDA data or DIA‐NN [59] (version 1.8.1) for DIA data. The database containing 3672 protein entries was downloaded from CyanoBase (ftp://ftp.kazusa.or.jp/pub/CyanoBase/*Synechocystis*; released on 2009‐05‐11).

### 4.12 Statistical Analysis

Bioinformatic and statistical analyses of proteomics data were conducted primarily with Perseus software (version 2.0.11.0) [60]. Student's *t*‐test was used to determine the significance of differential expression of proteins, and Fisher's‐exact test was used for the functional enrichment analysis. A *p*‐value < 0.05 was used as the cut‐off for all statistical analyses. DEPs were determined using thresholds of *p* < 0.05 and a fold change > 1.5. The gene ontology terms (GOBP, GOMF, and GOCC), KEGG pathways (KEGG), and functional categories annotated by the CyanoBase (CyanoBase) were included for the functional enrichment analysis. *p*‐value < 0.05 and the enrichment factor >1.5 were used as the cutoffs to include all enriched categories.

For other data analyses, all experiments were performed in triplicate (*n* = 3), and data are presented as the mean ± standard deviation (SD) unless otherwise indicated in the figure legends. No data points were excluded from the analyses. No statistical methods were employed to predetermine sample size for other experiments. Unless otherwise stated, all statistical tests were two‐sided, and results were considered statistically significant when the *p*‐value < 0.05. Statistical analyses were performed utilizing GraphPad Prism software (version 9.4.0). Statistical significance was defined as ^*^ *p* < 0.05, ^**^ *p* < 0.01, ^***^ *p* < 0.001, and ^****^ *p* < 0.0001.

## Author Contributions

W.Y. and Y.W. supervised and designed the study; C.H., Z.X., H.G., G.W., J.D., and H.Y. performed the experiments; C.H. and Z.X. analyzed the data; C.H., Z.X., H.G., G.W., J.D., Y.W., H.Y., X.W., Z.W., Y.Z., X.H., and Y.W. conducted the investigation; C.H., Z.X., W.Y., and Y.W. wrote the draft; W.X., W.Y., and Y.W. reviewed and edited the manuscript.

## Funding

The work was supported by Science and Technology Innovation 2030 Major Project (2024ZD0408004), National Key Research and Development Program of China (2022YFF1001704), and a grant from the National Natural Science Foundation of China (32170253).

## Conflicts of Interest

The authors declare no conflicts of interest.

## Supporting information




**Supporting File 1**: advs74770‐sup‐0001‐SuppMat.docx.

**Supporting File 2**: advs74770‐sup‐0002‐TableS1.xlsx.

**Supporting File 3**: advs74770‐sup‐0003‐TableS2.xlsx.

**Supporting File 4**: advs74770‐sup‐0004‐TableS3.xlsx.

**Supporting File 5**: advs74770‐sup‐0005‐TableS4.xlsx.

**Supporting File 6**: advs74770‐sup‐0006‐TableS5.xlsx.

**Supporting File 7**: advs74770‐sup‐0007‐TableS6.xlsx.

**Supporting File 8**: advs74770‐sup‐0008‐TableS7.xlsx.

## Data Availability

The mass spectrometry proteomics data have been deposited to the ProteomeXchange Consortium via the PRIDE [61] partner repository with the dataset identifier PXD066894. The data that support the findings of this study are available from the corresponding author upon reasonable request.
