# Supplementary material for: Coordination of Cyanobacterial Nitrate Assimilation and Photosynthesis by a Novel PsbO‐Interacting Protein PirN
Source: Adv Sci (Weinh). 2026 Mar 20;13(29):e18047. doi: 10.1002/advs.202518047 (PMC13205821; doi:10.1002/advs.202518047)
Supplement: Supplementary file 1 — Supporting File 1: advs74770‐sup‐0001‐SuppMat.docx. [file ADVS-13-e18047-s005.docx]

**Supplementary information for**

**Coordination of Cyanobacterial Nitrate Assimilation and Photosynthesis by a Novel PsbO-interacting Protein PirN**

*Chengcheng Huang*^1^, *Zhen Xiao*^1,2,3^, *Haitao Ge*^1^, *Gaojie Wang*^1,2^, *Jinghui Dong*^1,2^, *Yan Wang*^1,2^, *Hang Yang*^1,2^, *Xing Wang*^1,2^, *Hui Gao*^1,2^, *Zhongshu Wang*^1,2^, *Huanling Yang*^4^, *Yuanya Zhang*^1^, *Xiahe Huang*^1^, *Wu Xu*^5^, *Weimin Ma*^6^, *Wenqiang Yang*^2,4,7^*, and *Yingchun Wang*^1,2^*

^1^Institute of Genetics and Developmental Biology, Chinese Academy of Sciences, No.1 West Beichen Rd., Beijing 100101, China.

^2^University of Chinese Academy of Sciences, Beijing 100049, China.

^3^Present address: Central Laboratory, Shanxi Province Cancer Hospital/Shanxi Hospital Affiliated to Cancer Hospital, Chinese Academy of Medical Sciences/Cancer Hospital Affiliated to Shanxi Medical University, Taiyuan 030001, Shanxi, China.

^4^State Key Laboratory of Forage Breeding-by-Design and Utilization and Key Laboratory of photobiology, Institute of Botany, Chinese Academy of Sciences, Beijing 100093, China.

^5^Department of Chemistry, University of Louisiana at Lafayette, Lafayette, LA 70504, USA.

^6^College of life Sciences, Shanghai Normal University, 100 Guilin Road, Shanghai, China.

^7^China National Botanical Garden, Beijing 100093, China.

*Chengcheng Huang* and *Zhen Xiao* contributed equally to this work.

Correspondence: Yingchun Wang ([ycwang@genetics.ac.cn](mailto:ycwang@genetics.ac.cn)) Wenqiang Yang (wqyang@ibcas.ac.cn)

**
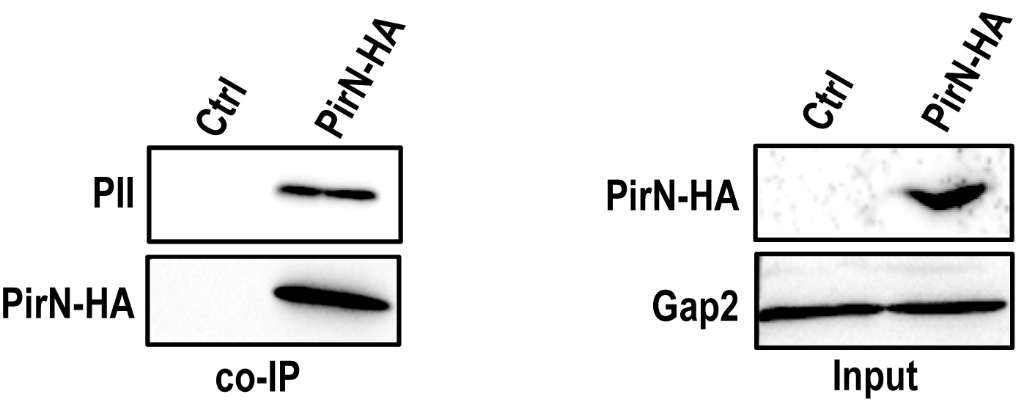
**

**Supplemental Figure S1.** **Confirmation of PirN-PII interaction by co-IP coupled with Western blotting.**

Whole-cell lysates derived from wild-type (WT, control) and PirN-HA knock-in strains of *Synechocystis* were subjected to co-immunoprecipitation (co-IP) assays using anti-HA and anti-PII antibodies, respectively. Equal amounts of input samples were also probed for Gap2, which served as a loading control.

**
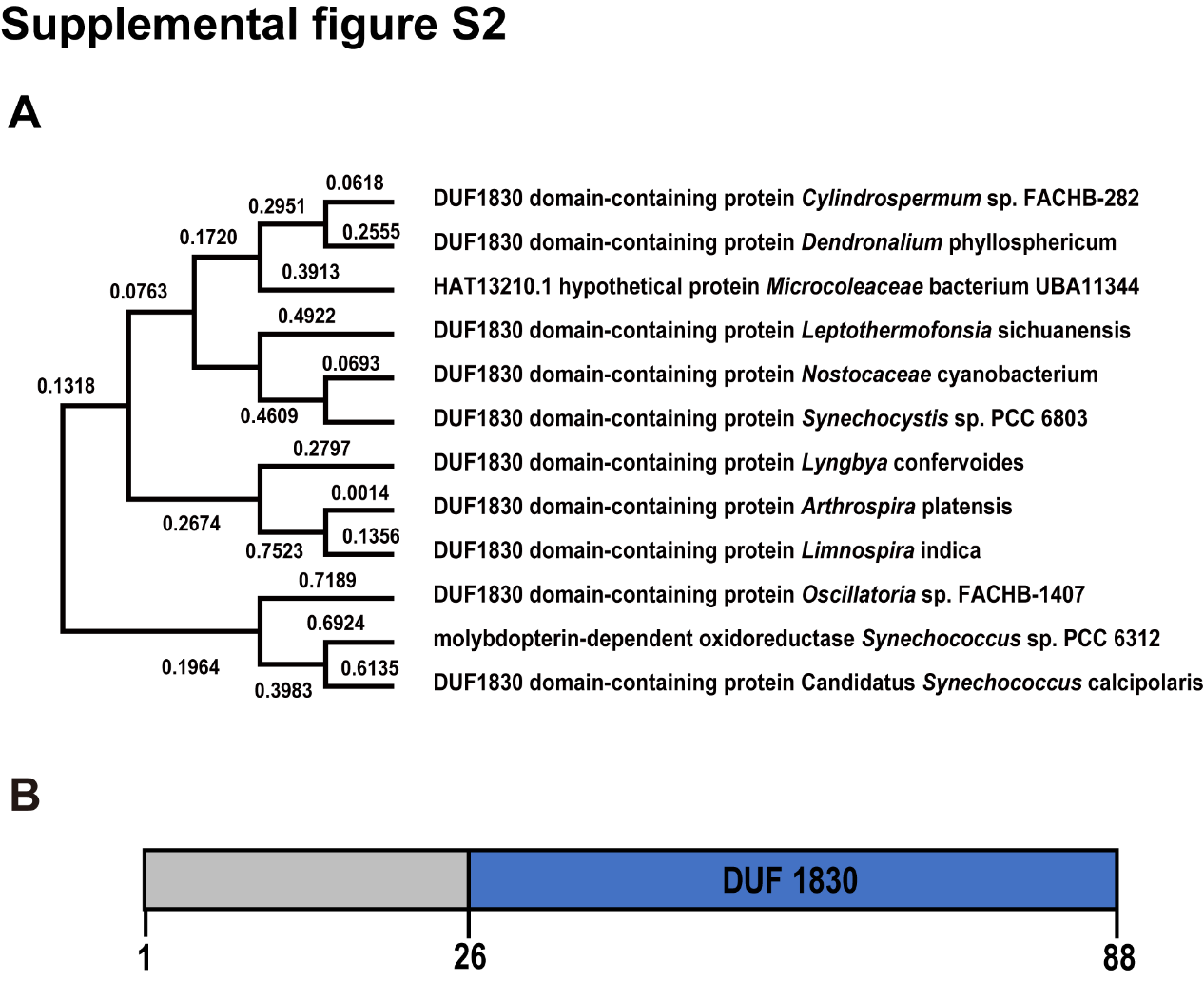
**

**Supplemental Figure S2.** **Phylogenetic and domain analysis of PirN.**

(**A**) Phylogenetic tree of PirN and its homologs in cyanobacteria, constructed using MEGA11 software [1]. Protein sequences were obtained from the NCBI database ([www.ncbi.nlm.nih.gov](http://www.ncbi.nlm.nih.gov)).

(**B**) Schematic representation of PirN highlighting predicted Pfam domains.

**
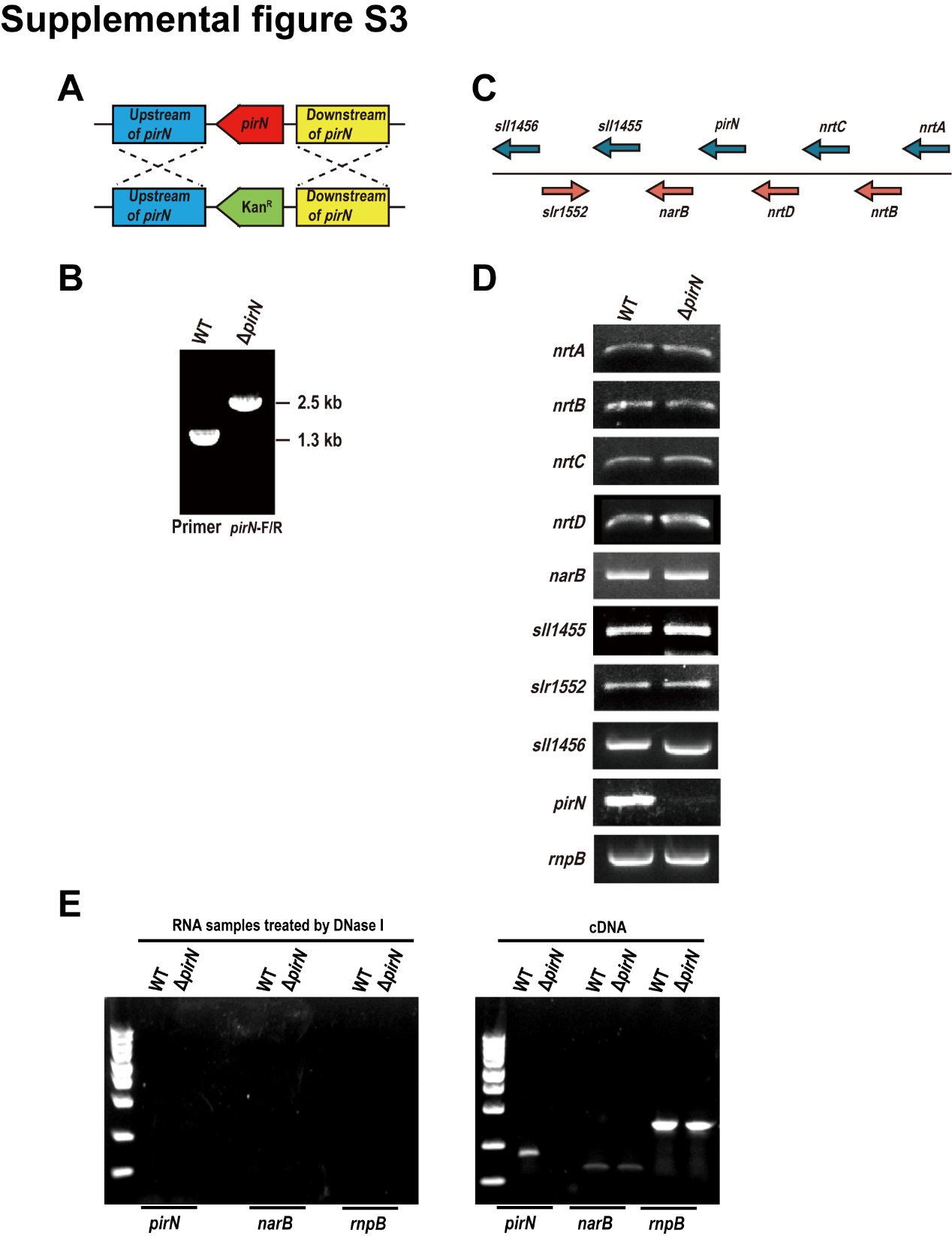
**

**Supplemental Figure S3. Generation and validation of *pirN*-deletion mutant (Δ*pirN*).**

(**A**) Schematic diagram of the plasmid construct used to delete the *pirN* open reading frame (ORF) from the *Synechocystis* genome. Kan^R^: Kanamycin resistance cassette.

(**B**) PCR confirmation of complete segregation of Δ*pirN* mutant. The same primer pair was used to amplify genomic DNA from both WT and mutant strains.

(**C**) Schematic representation of the genomic region containing *pirN* and its neighboring ORFs.

(**D**) RT-PCR detection of transcript levels for *pirN* and its neighboring ORFs in WT and Δ*pirN* mutant. *rnpB* was used as the internal loading control.

(**E**) PCR assay to assess potential residual genomic DNA (gDNA) contamination in RNA samples from the WT and Δ*pirN* mutant.


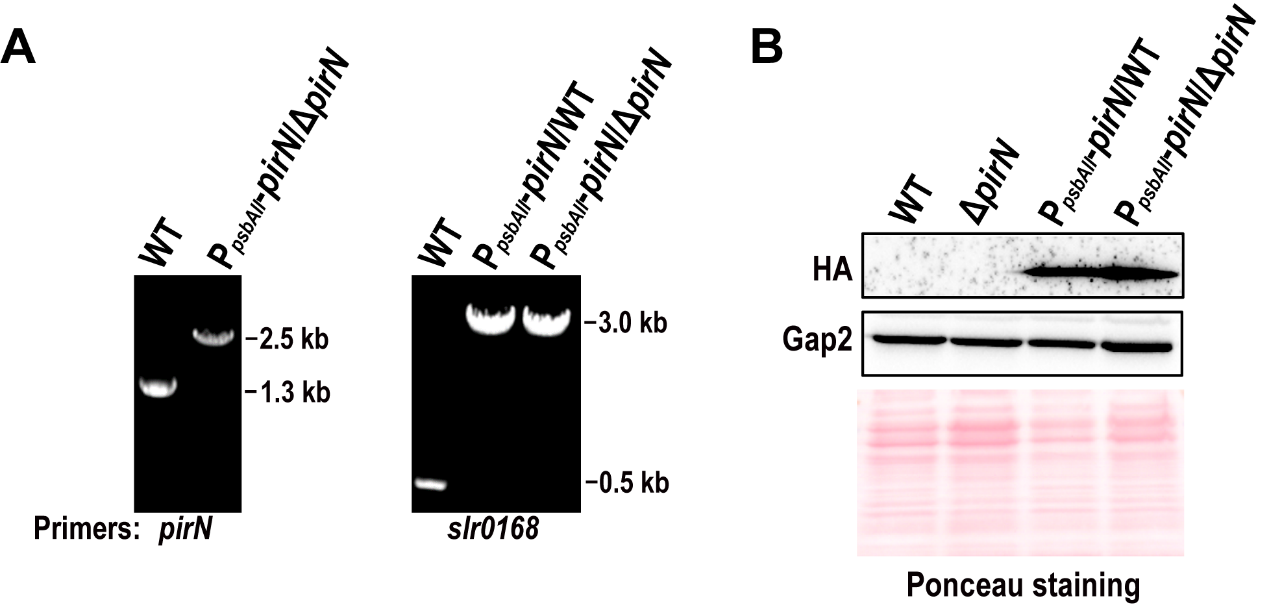


**Supplemental Figure S4. Confirmation of the PirN-complemented strains.**

(**A**) PCR verification of complete segregation of the indicated mutant strains.

(**B**) Western blot analysis confirming PirN expression in the PirN-complemented strains. Gap2 blot and Ponceau S staining were used as loading controls.

**
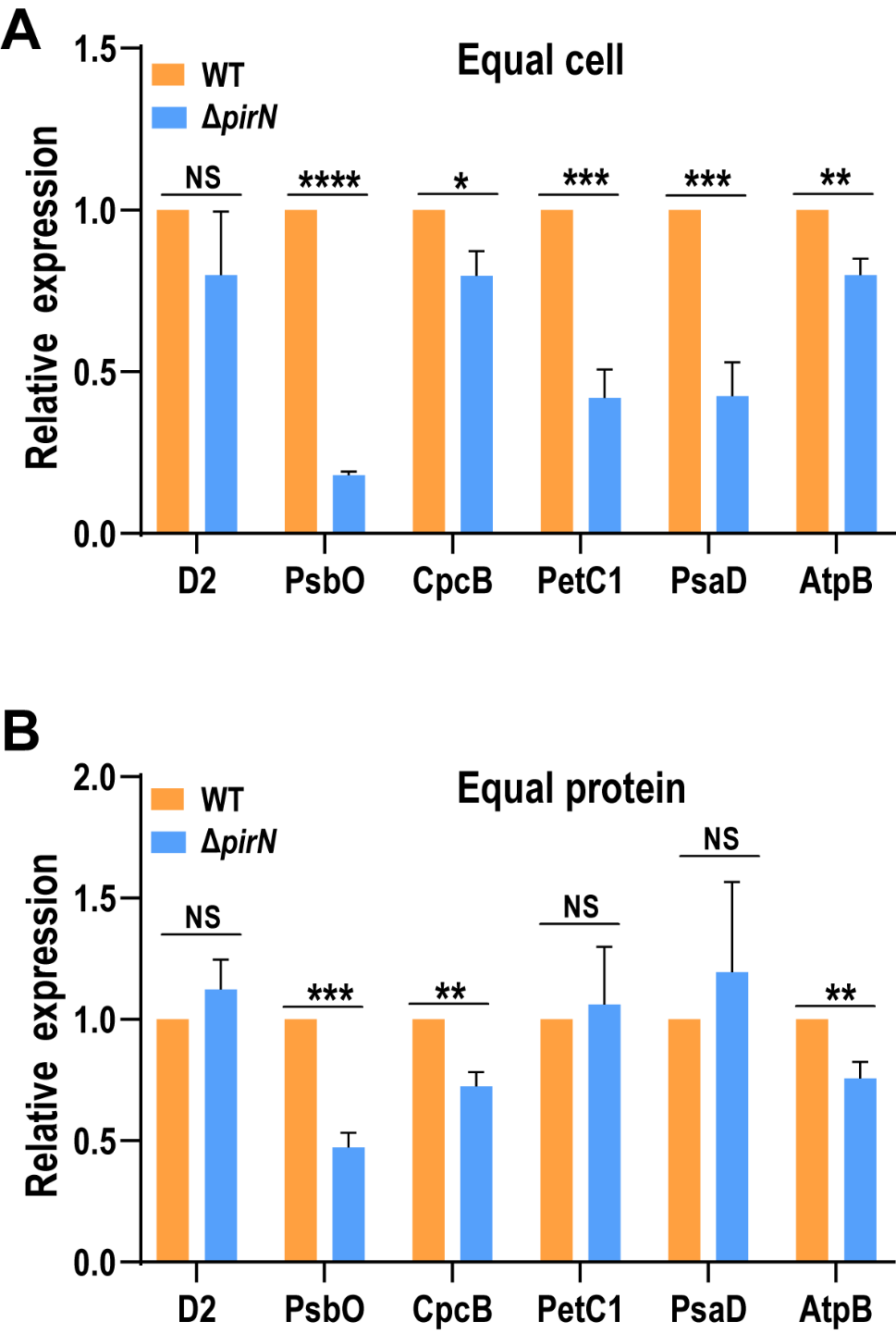
**

**Supplemental Figure S5. Quantification of representative photosynthesis related proteins by Western blotting and densitometric analysis.**

The Western-blot results are shown in Figure 2D, and were quantified on a per cell **(A)** and per protein **(B)** basis. Protein expression levels in each mutant were quantified relative to those in the WT using ImageJ software. Data are presented as the mean ± SD from three biological replicates (n = 3). Statistical significance was determined using Student’s *t*-test. NS: not significant, ****: *p* < 0.0001, ***: *p* < 0.001, **: *p* <0.01, *: *p* < 0.05.

**
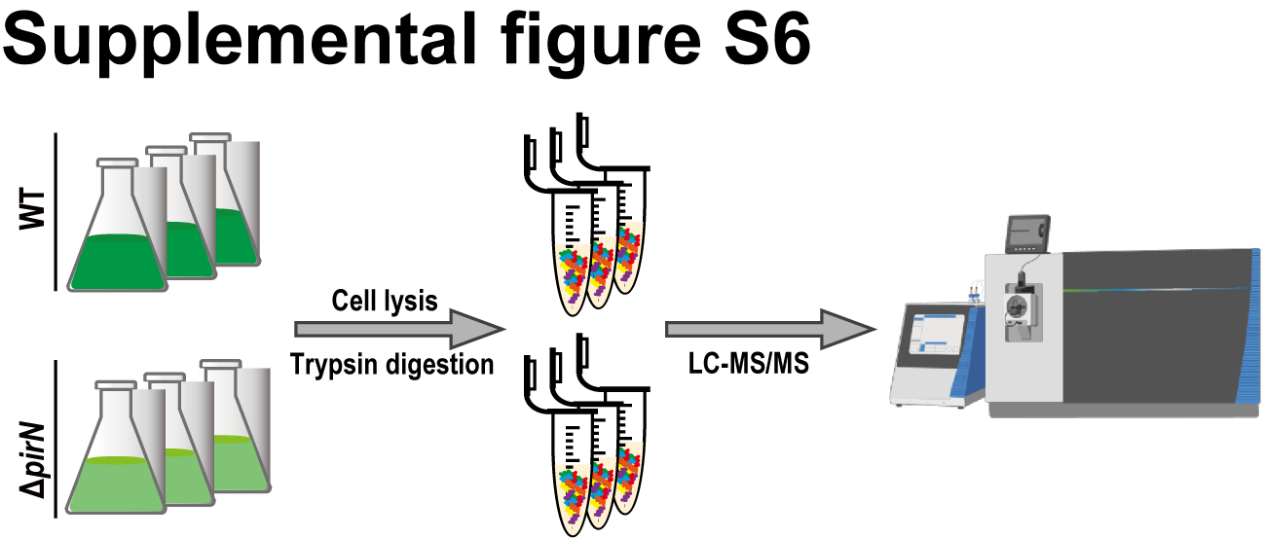
**

**Supplemental Figure S6. Schematic diagram illustrating the workflow of quantitative proteomic analysis for the Δ*pirN* mutant.**

**
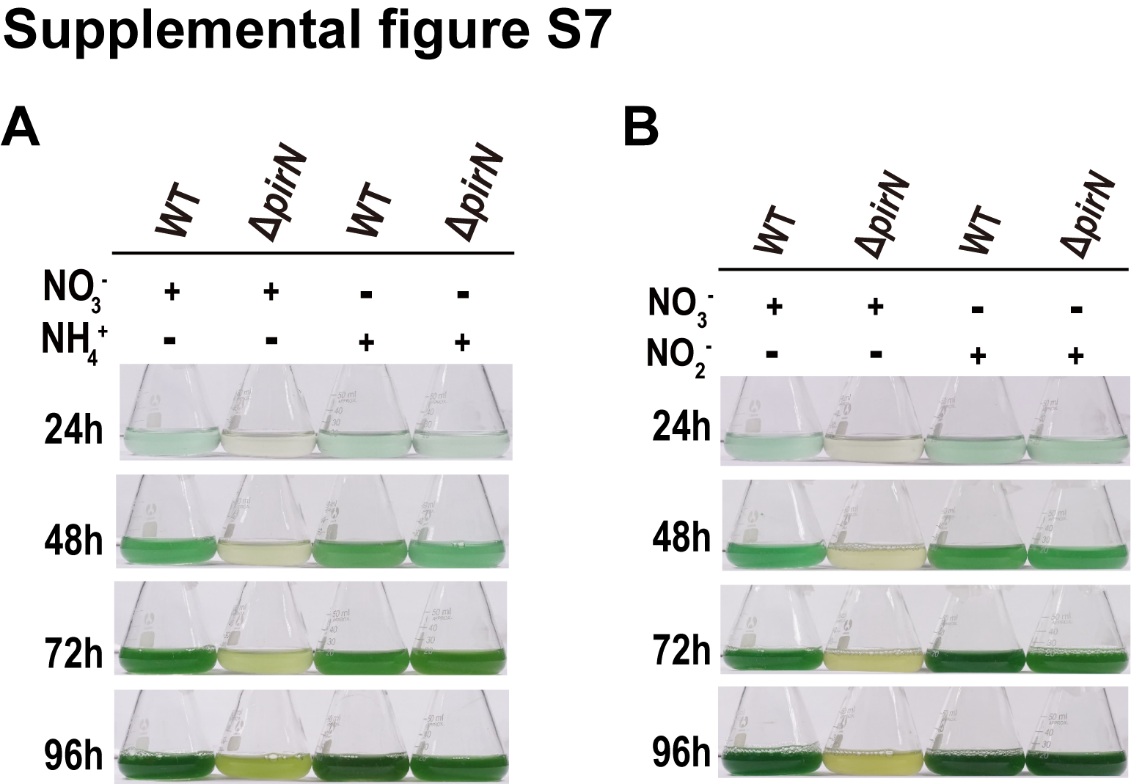
**

**Supplemental Figure S7. Growth experiments of the WT and Δ*pirN* under different nitrogen conditions.**

WT and Δ*pirN* cells were cultured in liquid BG-11 medium supplemented with either ammonium (**A**), nitrite (**B**), or nitrate (control) as the sole nitrogen source. Cultures were photographed every 24 h to monitor changes in cell density and pigmentation.

**
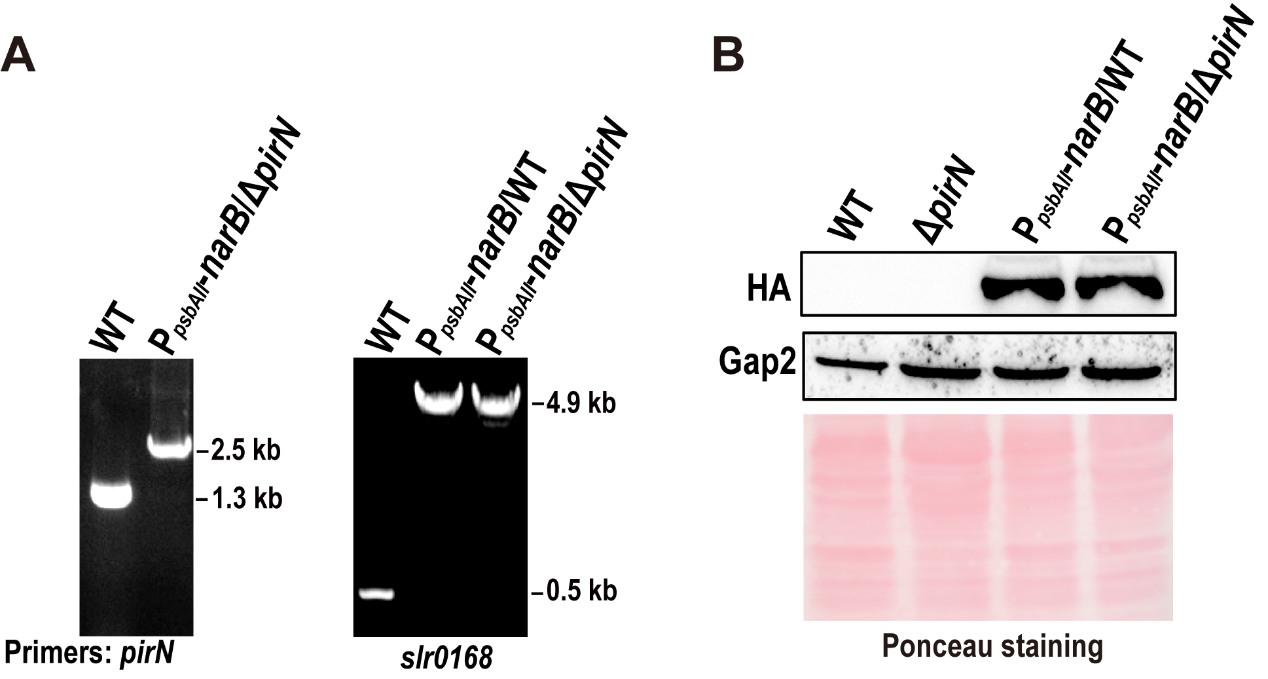
**

**Supplemental Figure S8. Generation and validation of NarB-complemented *Synechocystis* strains.**

**(A)** PCR confirmation of the complete segregation of the indicated mutants

**(B)** Western blot analysis confirming NarB expression in the NarB-complemented strains.


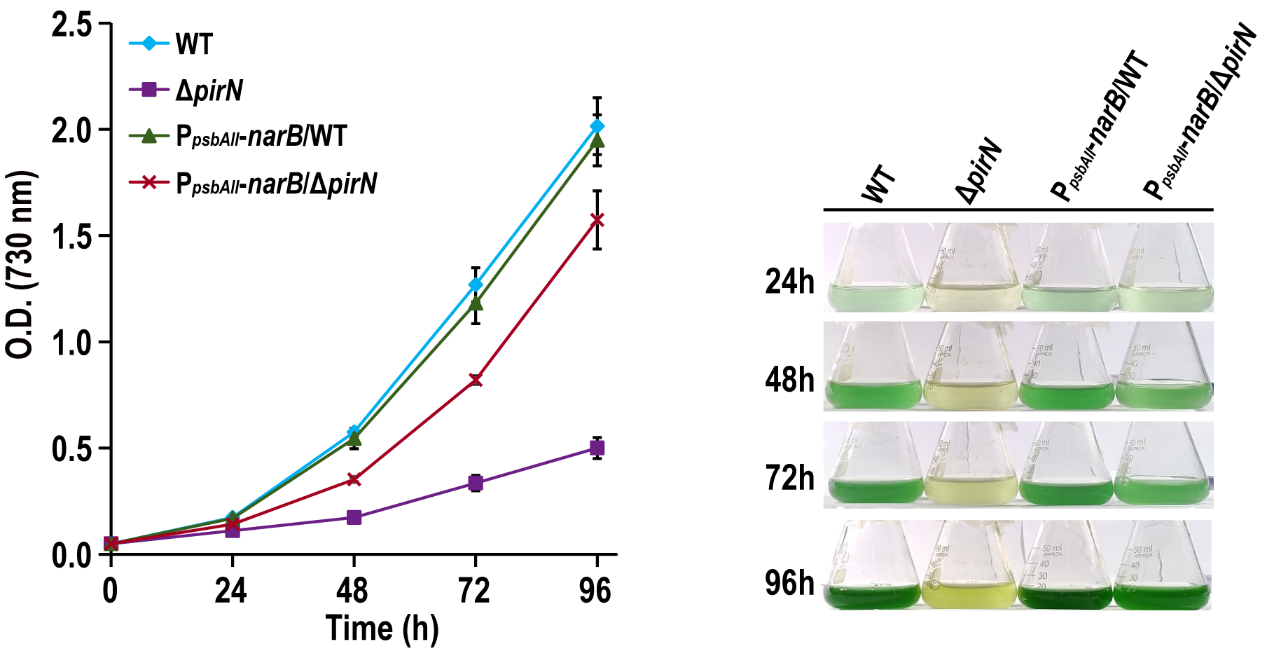


**Supplemental Figure S9. Growth experiments of the WT and mutant strains under photoautotrophic conditions.**

Growth curves of the indicated strains under photoautotrophic conditions (left panel). Data are presented as the mean ± SD from three biological replicates (n = 3). The cultures were photographed every 24 h to monitor cell density and color changes (right panel).

**
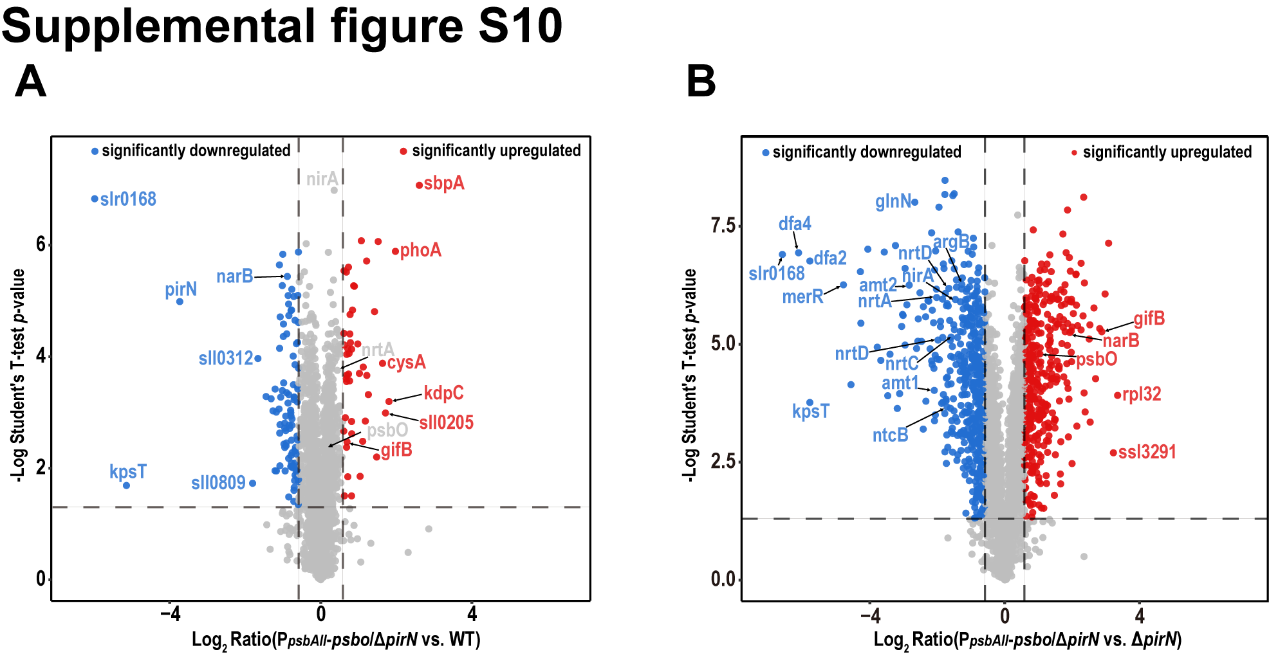
**

**Supplemental Figure S10. Volcano plots showing differentially expressed proteins under the indicated experimental conditions.**

**(A)** P*_psbAII_*-*psbO*/Δ*pirN* vs. WT.

**(B)** P*_psbAII_*-*psbO*/Δ*pirN* vs. Δ*pirN*. The dashed lines indicate the thresholds for fold change (vertical) and *p*-value (horizontal).


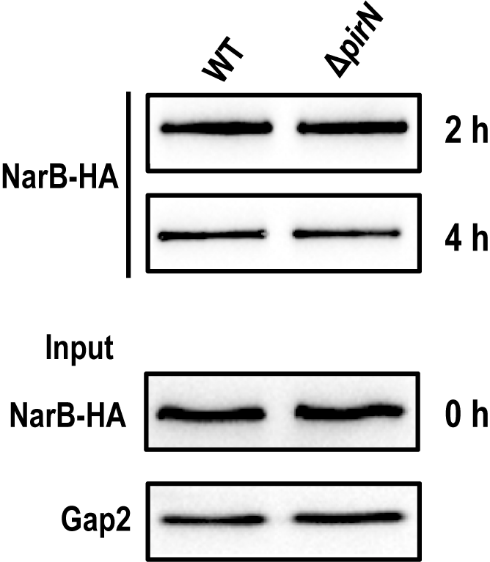


**Supplemental Figure S11. In vitro stability assay of NarB-HA.**

NarB-HA immunoprecipitated from *Synechocystis* knock-in strain expressing NarB-HA was separately incubated with lysates from WT and Δ*pirN* strains for the indicated time durations, and then detected by Western blot using anti-HA antibody. A fraction of NarB-HA before incubation and Gap2 in the lysates were also probed as the loading control.


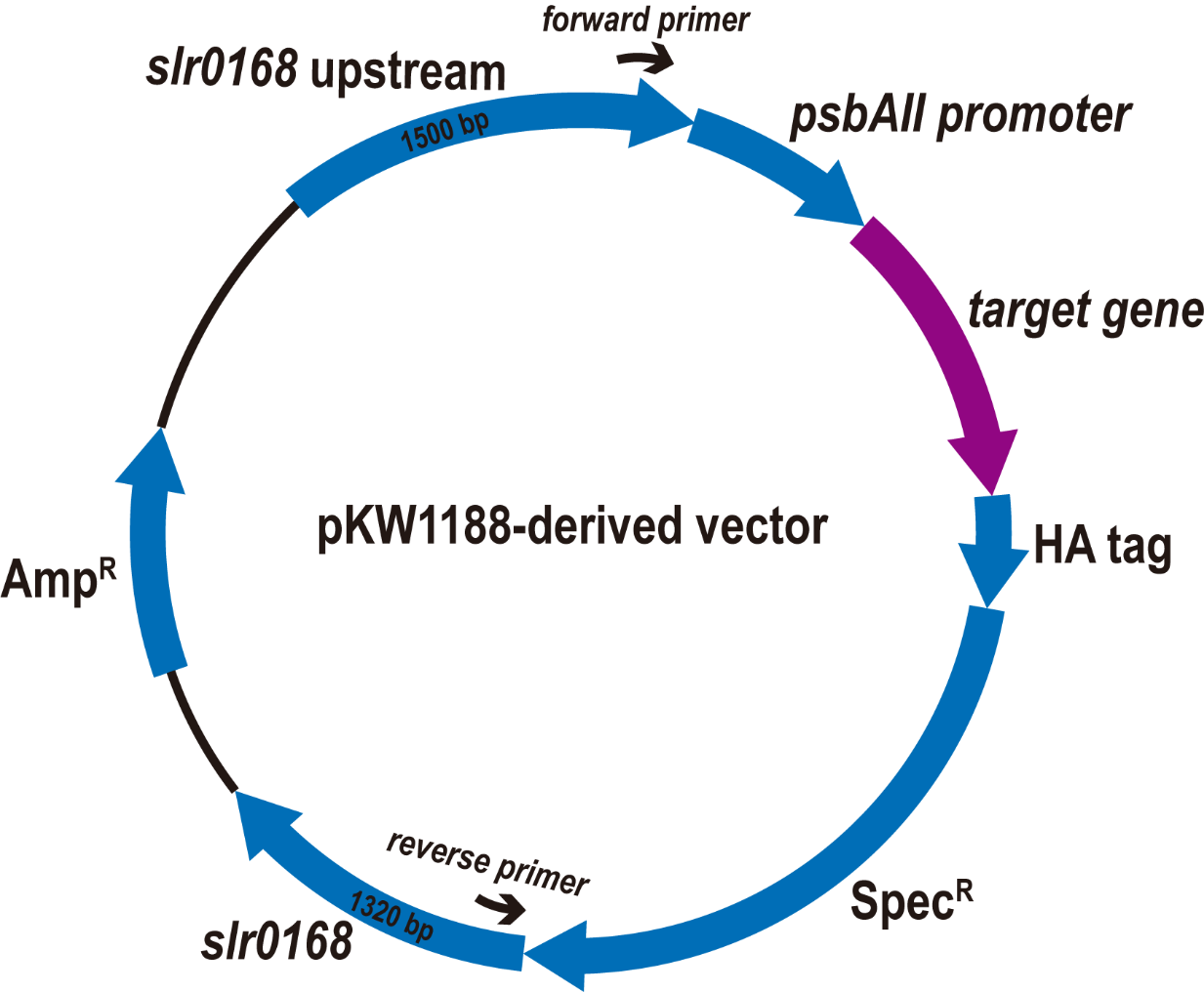


**Supplemental Figure S12. Schematic diagram of the recombinant vector structure.**

Spec^R^: Spectinomycin resistance gene cassette. Amp^R^: Ampicillin resistance gene cassette.

**Reference**

[1] K. Tamura, G. Stecher, S. Kumar, "MEGA11: Molecular Evolutionary Genetics Analysis Version 11," *Mol Biol Evol (2021):* 38 (7), 3022, <https://doi.org/10.1093/molbev/msab120>.
